# CRISPR-based m^6^A modification and its potential applications in telomerase regulation

**DOI:** 10.3389/fcell.2023.1200734

**Published:** 2023-07-14

**Authors:** Mingliang Yi, Mingyue Wang, Yongjie Xu, Zhikun Cao, Yinghui Ling, Zijun Zhang, Hongguo Cao

**Affiliations:** ^1^ Anhui Province Key Laboratory of Local Livestock and Poultry Genetic Resource Conservation and Bio-breeding, Anhui Agricultural University, Hefei, China; ^2^ College of Animal Science and Technology, Anhui Agricultural University, Hefei, China

**Keywords:** CRISPR system, m^6^A modification, epigenetic regulation, telomere, telomerase

## Abstract

Telomerase determines cell lifespan by controlling chromosome stability and cell viability, m^6^A epigenetic modification plays an important role in the regulation of telomerase activity. Using CRISPR epigenome editing to analyze specific m^6^A modification sites in telomerase will provide an important tool for analyzing the molecular mechanism of m^6^A modification regulating telomerase activity. In this review, we clarified the relevant applications of CRISPR system, paid special attention to the regulation of m^6^A modification in stem cells and cancer cells based on CRISPR system, emphasized the regulation of m^6^A modification on telomerase activity, pointed out that m^6^A modification sites regulate telomerase activity, and discussed strategies based on telomerase activity and disease treatment, which are helpful to promote the research of anti-aging and tumor related diseases.

## 1 Introduction

The research of telomere and telomerase is of great significance to the aging of organism. Telomerase is an eukaryotic ribonucleoprotein (RNP) composed of RNA-protein complex (Blackburn and Collins, 2011). It extends the 3‘end of linear chromosome by synthesizing the telomere repeat TTAGGG to maintain telomere length and chromosome stability (Liu et al., 2022). Telomerase activity is closely related to tumorigenesis (Shay, 2016; Trybek et al., 2020), cell proliferation and cell aging (Chakravarti et al., 2021). N6-methyladenosine (m^6^A) RNA modification is an important epigenetic modification mode in post-transcriptional regulation (Schmidt et al., 1975; Roundtree et al., 2017a; Tang et al., 2020), which involves almost all aspects of RNA metabolism and affects various physiological and pathological processes by regulating mRNA cytoplasmic transport, splicing, stability, structure and translation (Dominissini et al., 2012). The composition of telomerase fully shows that it has a close relationship with m^6^A modification. The study of the mechanism of m^6^A modification regulating telomerase activity and maintaining telomere length will promote human anti-aging to provide new ideas.

With the application and improvement of CRISPR (clustered regularly interspaced short palindromic repeats)/Cas system (Jinek et al., 2012; Cong et al., 2013), the modified fusion protein dm^6^ACRISPR system can achieve precise and efficient m^6^A site-specific modification in RNA transcripts (Liu et al., 2019; Wilson et al., 2020), this will help to further explore the mechanism of m^6^A modification. In clinical cancer research, it was found that there was a site mutation in the promoter region of telomerase reverse transcriptase (TERT) gene. Use the base editing function of CRISPR system to reduce the transcription and protein expression of TERT, and induce the aging and proliferation stagnation of cancer cells, which verifies the feasibility of activated TERT promoter mutation as a cancer-specific therapeutic target (Killela et al., 2013; Li et al., 2020). Therefore, the CRISPR system technology, combined with the m^6^A modification of RNA and the regulation of telomerase activity, is used to regulate the aging and proliferation of cells in the body and achieve the treatment of various diseases.

In this review, we reviewed and discussed the latest research progress, and found that the CRISPR system was used to carry out m^6^A site-specific modification of RNA, regulate telomerase activity and affect telomere length by regulating telomerase assembly and other processes, which provided a direction for the study of epigenetic modification to regulate cell aging mechanism, and provided a prospect for the future research on cell proliferation and aging.

## 2 CRISPR system introduction

**FIGURE 1:**
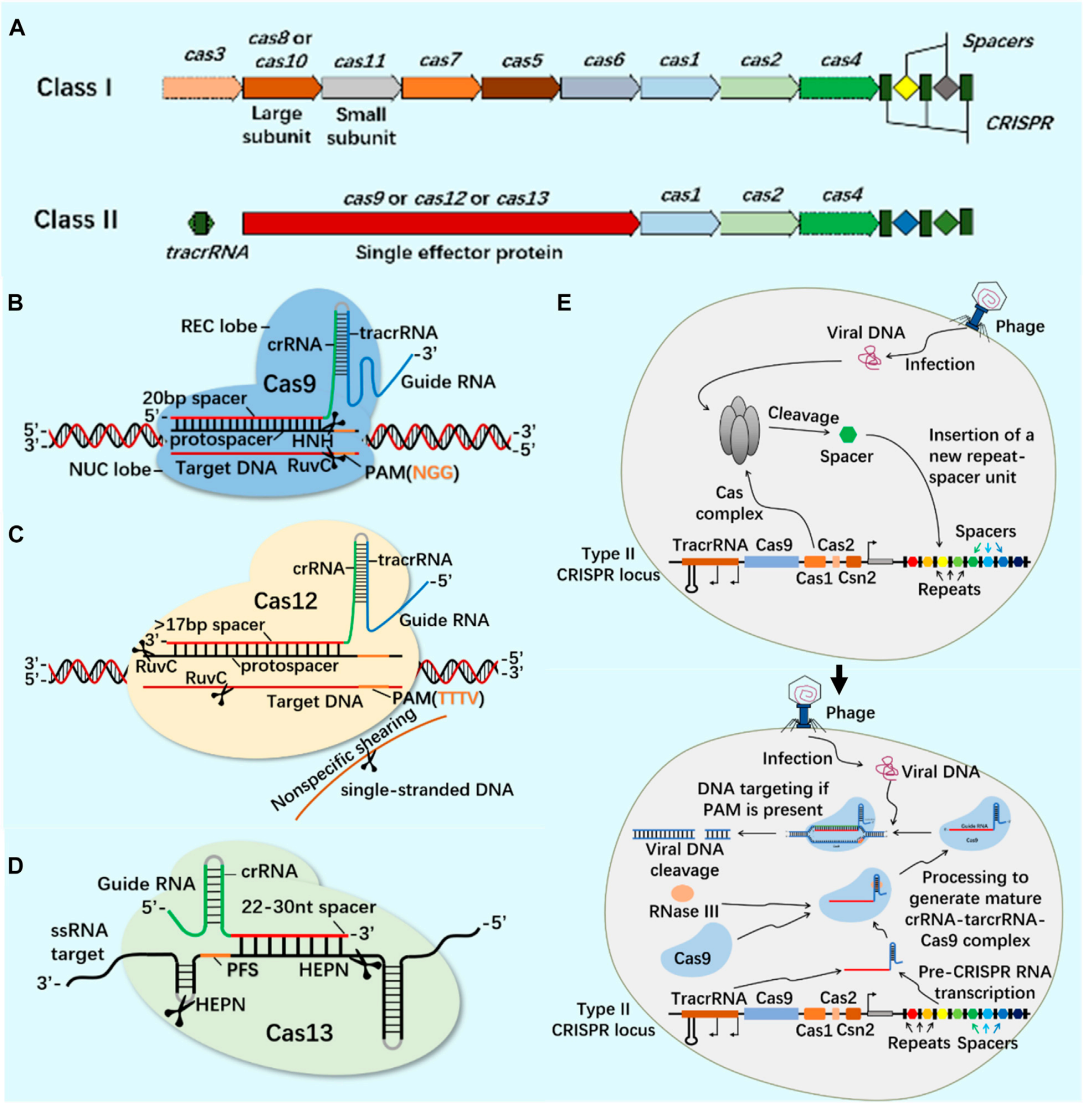
CRISPR/Cas system classification, typical structure, important members of Class II CRISPR/Cas system and principle of CRISPR/Cas system. **(A)** The structure of class I and class II CRISPR/Cas system loci. The class I of CRISPR/Cas system is composed of multiple Cas proteins to form a crRNA complex, which plays a role in the binding and processing of targets; The class II of CRISPR/Cas system is composed of a single multi-domain crRNA-binding protein, and its function is similar to the effect complex in the class I of CRISPR system. **(B)** CRISPR/Cas9 system is mainly composed of Cas9 protein and single-stranded guide RNA (sgRNA). Cas9 protein has the function of cutting DNA double-stranded, and sgRNA plays a guiding role. In the presence of the adjacent motif (PAM) of the prototype spacer, Cas9 protein can reach different target sites through base complementary pairing under the guidance of sgRNA, and achieve DNA double strand break (DSB) by cutting the target gene through the two nuclease domains of RuvC and HNH. **(C)** CRISPR/Cas12 system is mainly composed of Cas12 protein and single-stranded guide RNA (sgRNA). Cas12 protein has side-cutting activity and can cut double and single strands of DNA. It mainly plays the role of inducing DSBs through a single RuvC-like nuclease domain. **(D)** CRISPR/Cas13 system is mainly composed of Cas13 protein and single-stranded guide RNA (sgRNA). Cas13 protein is a single protein composed of multiple domains. It has the function of recognizing crRNA, cutting RNA, and even cutting pre-crRNA. It has the phenomenon of side-cutting activity similar to CRISPR/Cas12 system. It cuts RNA through two HEPN domains. **(E)** CRISPR/Cas system is an acquired immune system from bacteria and archaea. Taking the principle of CRISPR/Cas9 system as an example: the acquisition of highly variable spacer of CRISPR - the expression of CRISPR loci (transcription and post-transcriptional maturation) - the development of CRISPR/Cas9 system activity.

CRISPR/Cas9 system widely exists in prokaryotes and provides acquired immunity against the invasion of foreign viruses and plasmids (Ghaemi et al., 2021). The developed CRISPR/Cas system can accurately edit DNA or RNA targets at specific sites and has been widely used in gene editing (Hsu et al., 2014; Manghwar et al., 2019; Zhang et al., 2021). According to the composition of Cas effector proteins, CRISPR system is divided into Class I and Class II (Figure 1). These systems use site-specific guide RNA to guide Cas protein and accurately edit site-specific sequences (Wang et al., 2022; Yu et al., 2022). At present, the most widely used CRISPR systems are Class II Type II Cas9, Type V Cas12 and Type VI Cas13 (Figure 1).

The CRISPR system is mainly used to modify specific target genes in the genome of organisms. The main editors include DNA cytosine base editor (CBE), adenine base editor (ABE) and primer editor (PE) (Kantor et al., 2020). PE is a multi-functional and high-precision genome editor (Anzalone et al., 2019), which is composed of two parts: the leader editor protein and primer editing guided RNA (pegRNA). Using the CRISPR Cas protein targeted DNA to make nicks and the DNA synthesis ability of reverse transcriptase, the sequence encoded by pegRNA can be accurately and efficiently copied into the targeted DNA sequence to achieve accurate editing, including replacement, insertion and deletion (Anzalone et al., 2019; Kantor et al., 2020; Kim et al., 2020). The advantage of PE is that it does not cause DNA double strand breakage, only cutting one strand of DNA, thereby avoiding potential risks such as chromosome loss and rearrangement caused by double strand DNA breakage. Researchers can further improve the accuracy and specificity of PE by optimizing lead editing proteins, pegRNA, and AAV genomic elements, such as introducing engineered Cas9 mutants, especially eSpCas9 and Sniper Cas9 mutants, into PE (Kim et al., 2020). The PE editing efficiency prediction models DeepPrime, DeepPrime FT, and the off target prediction model DeepPrime Off make the design and screening of pegRNAs more convenient and efficient, providing strong guarantees for the future widespread application of PE systems (Yu et al., 2023). Using PE to repair sickle cell anemia (SCD) mutations in hematopoietic stem cells or progenitor cells of patients, the repaired cells are treated for hereditary blood diseases through transplantation (Everette et al., 2023). The gene editing system developed based on CRISPR technology has brought prospects for the research and treatment of genetic diseases.

## 3 CRISPR system and RNA editing

**FIGURE 2:**
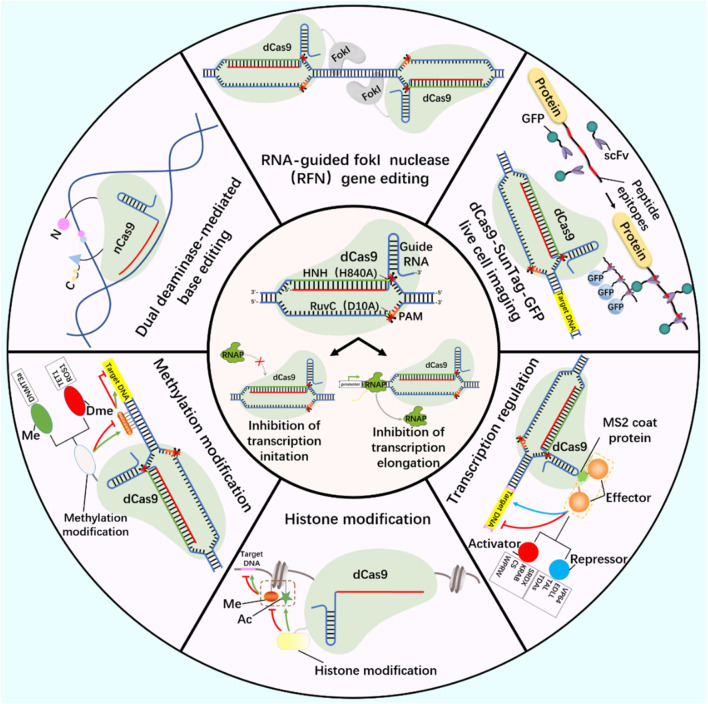
DNA editing tools-CRISPR/dCas9.

**FIGURE 3:**
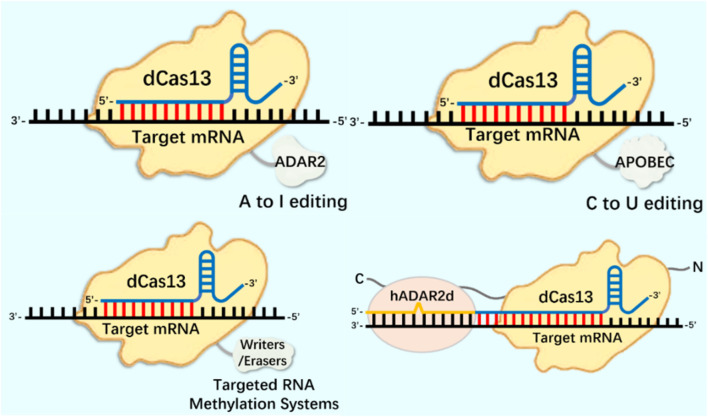
CRISPR system in RNA editing applications.

At present, CRISPR/Cas9 and CRISPR/Cas13 systems have become tools for the research and application of DNA and RNA epigenetic modification (Figure 2; Figure 3) (Zhan et al., 2019; Kordyś et al., 2022). Nucleic acid endonuclease deficient Cas9 (dCas9)/Cas13 (dCas13) still has the activity of binding enzyme, which can combine with effector protein to regulate the expression of DNA or RNA, becoming an effective method to study gene function and regulation mechanism (Yang et al., 2019; Liu et al., 2020).

Working principle of CRISPR/dCas9 system and related tools based on CRISPR/dCas9 system development (gene editing, live cell imaging, base editing, methylation modification, histone modification and transcription regulation). Ac, acetylation; Me, Methylation; Dme, Demethylation.

### 3.1 RNA editing tools: based on CRISPR/dCas13 system

The modification of CRISPR system for gene editing at the DNA level is irreversible, especially the ethical issues involved in the safety of human germ cell and embryonic cell editing cannot be ignored (Leibowitz et al., 2021; Höijer et al., 2022). p53 gene is a tumor suppressor gene that participates in the regulation of cell growth, differentiation, apoptosis and other processes (Fischer et al., 2016). After Cas9 protein was introduced into cells to realize CRISPR/Cas9 mediated genome editing, p53 pathway was upregulated and DNA repair level was increased. Cas9 protein induces p53 pathway activation and p53 mediated DNA damage response (Enache et al., 2020). These findings are of great significance for the correct application of CRISPR/Cas9 mediated genome editing (Enache et al., 2020; Sinha et al., 2021). Therefore, the use of CRISPR/Cas9 technology in human pluripotent stem cells (hPSCs) cell replacement therapy should be carefully carried out and the p53 function of hPSCs cells should be monitored (Ihry et al., 2018). The modification of Cas13 protein at the RNA level successfully avoids irreversible permanent changes to the genome, and is an important tool for studying the most abundant m^6^A modification on RNA. At the same time, it plays an important role in studying the structure and function of telomerase composed of RNA and protein.

In terms of RNA editing, the CRISPR system has been deeply modified and applied to mRNA epigenetic modification research. The m^1^A modification detection method based on the CRISPR/Cas13a system has been successfully used to identify m^1^A in 28S rRNA (Chen et al., 2019). The catalytic inactivation of RfxCas13d (dCasRx) is fused with the m^1^A demethylase ALKBH3, and the dCasRx ALKBH3 fusion protein can mediate effective demethylation of m^1^A modified RNA, known as Reengined m^1^A modification valid eraser (“REVER”), providing a tool for further elucidating the relationship between m^1^A modification of specific transcripts and their phenotypic results (Xie et al., 2021). m^1^A regulates the level of glycolysis in tumor cells by regulating the expression of ATP5D in the mitochondrial ATP synthase F1 domain. The dm^1^ACRISPR system can upregulate the expression of ATP5D through targeted removal of ATP5D m^1^A modification, resulting in an increase in the level of glycolysis of tumor cells (Wu et al., 2022). This is similar to using the CRISPR system to study m^6^A modification, where endogenous editing studies can be conducted by identifying the targets of epigenetic modifications on mRNA such as m^1^A and m^5^C. Because the CRISPR/RfxCas13d (CasRx) related transcriptome epigenetic modification editor has the characteristics of small size and high editing efficiency (Konermann et al., 2018; Zhang et al., 2018), which is suitable for packaging into lentivirus vector for gene function research. At present, CasRx has been successfully used to knock down specific mRNA transcripts in zebrafish embryos (Kushawah et al., 2020), and to mediate RNA targeted treatment of age-related macular degeneration in model mice (Zhou et al., 2020).

## 4 CRISPR system and m^6^A modification

**FIGURE 4:**
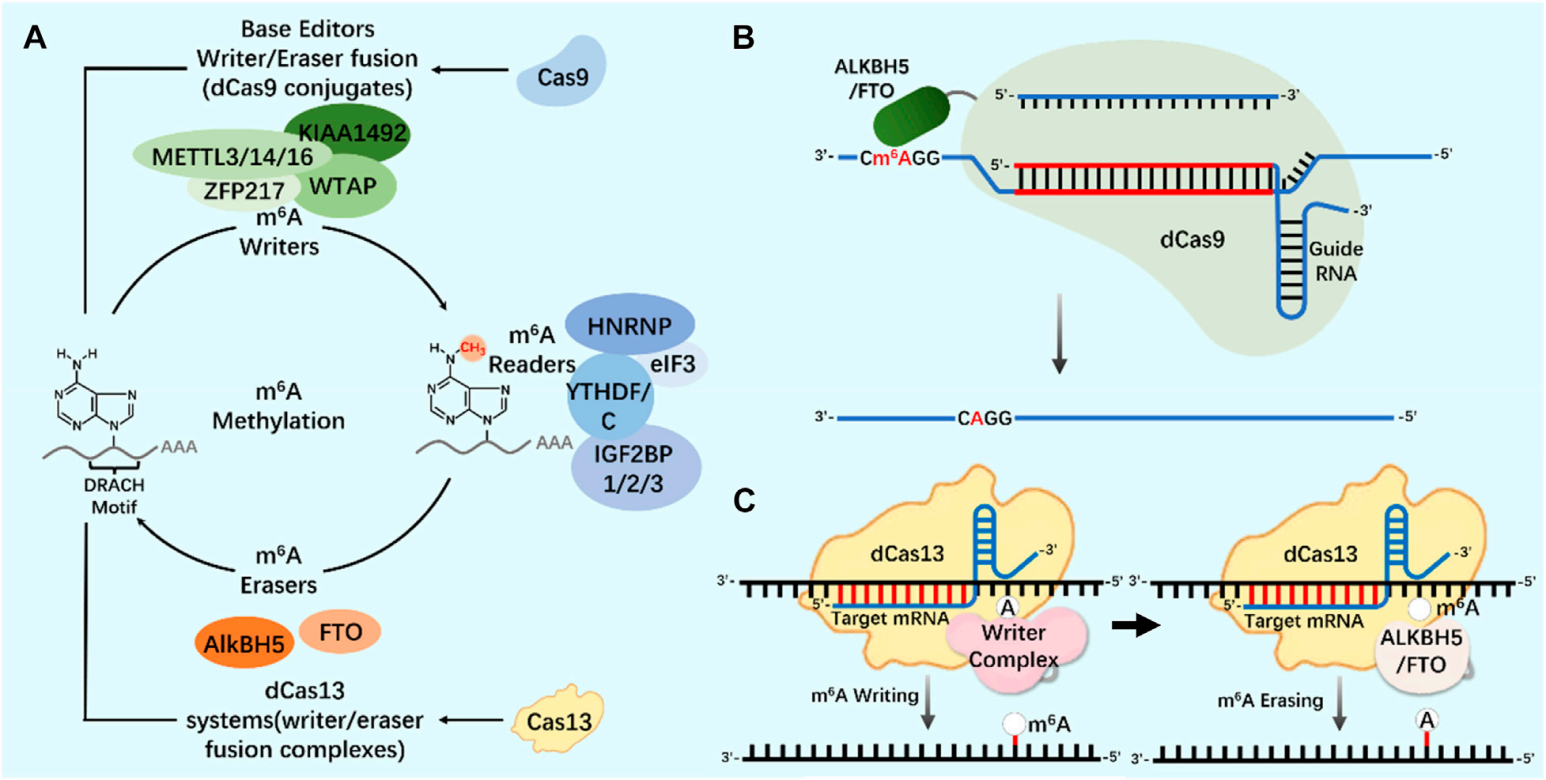
CRISPR system and m^6^A modification **(A)** N6-methyladenosine (m^6^A) regulation mechanism. Writers and erasers strictly regulate the presence of m^6^A on transcripts by targeting the m^6^A motif (DRACH). m^6^A is recognized by readers and starts the steps of regulating mRNA stability and translation. The modification system can be extended to include Cas9 (base editor, writer/eraser fusion) and Cas13 (methylation system). **(B)** Application of CRISPR/dCas9-ALKBH5/FTO tool in m^6^A modification. **(C)** Application of CRISPR/dCas13-Writer Complex tool in m^6^A modification; Application of CRISPR/dCas13-ALKBH5/FTO tool in m^6^A modification.

As an important biological function of RNA modification, m^6^A modification widely exists in almost all types of RNA molecules in cells (Yang et al., 2018; Hu et al., 2022). In the regulation of m^6^A modification, combining the modified protein specific domain with the inactivated CRISPR protein can produce a new precise editing tool for RNA methylation modification (Figure 4) (Li et al., 2020; Wilson et al., 2020; Kordyś et al., 2022). Liu *et al.* designed m^6^A modified eraser by combining CRISPR/Cas9 with demethylase ALKBH5 or FTO to realize RNA site-specific demethylation (Liu et al., 2019). Considering the important regulatory role of m^6^A modification on RNA in the nucleus, based on the RNA-targeted endonuclease system CRISPR/Cas13, an editor for targeted RNA methylation (TRM) was constructed, which became a new accurate editing tool for m^6^A modification. The editor can achieve efficient and accurate editing of m^6^A modification of RNA in nucleus and cytoplasm through nuclear export-signal (NES) and nuclear localization signal (NLS) (Wilson et al., 2020). The dm^6^ACRISPR editing tool can realize m^6^A modification of RNA sites, providing a more powerful weapon for in-depth research on the function of m^6^A modification (Table 1).

**TABLE 1:** Application of CRISPR system associated key regulatory factors in m^6^A modification.

CRISPR system	m^6^A key factor	Function	Year [Ref]
CRISPR/Cas9	METTL3	Promotes the increase of telomerase activity	2021 Lee et al. (2021)
		Mediates CDCP1 mRNA specific m^6^A installation to promote BC development	2020 Ying et al. (2020)
	ALKBH5	Significantly reduce methylation by targeting A2577 site with sgRNA	2019 Liu et al. (2019)
	FTO		
	METTL3-METTL14 heterodimer	Catalyzes 5‘UTR to increase m^6^A modification	
CRISPR/Cas13	ALKBH5	Promotes the stability of mRNA	2021 Li et al. (2020a)
		Accurate and reversible demethylation of targeted m^6^A sites of mRNA	2021 Chen et al. (2021)
		Mediates specific demethylation of m^6^A site to adenosine	2021 Xia et al. (2021)
	METTL3	Improves the modification efficiency of m^6^A in endogenous RNA transcripts	2020 Wilson et al. (2020)
		Mediates m^6^A specific methylation of adenosine sites	2021 Xia et al. (2021)
	YTHDF3	Inhibition of melanoma metastasis by interfering with YTHDF3-LOXL3 axis	2022 Shi et al. (2022)
	METTL3-METTL14 methyltransferase complex	Targeting m^6^A modification of exogenous RNA sites	2020 Wilson et al. (2020)
	FTO	Mediates m^6^A demethylation of long-interspersed element-1 (LINE1) RNA, regulating LINE1 RNA abundance and the local chromatin state	2022 Wei et al. (2022)

## 5 CRISPR system and telomerase

Telomerase, as an enzymatic RNP complex, plays a role of reverse transcriptase in the process of telomere elongation, and is significantly associated with cell aging and tumorigenesis (Sun et al., 2019). In cancer cells (Bajaj et al., 2020; Negrini et al., 2020; Wu et al., 2020), hematopoietic stem cells (Celtikci et al., 2021) and germ cells (Dogan and Forsyth, 2021; Lupatov and Yarygin, 2022), telomerase showed high activity (Demanelis et al., 2020). Cancer is closely related to a series of changes in intracellular genome and epigenome (Ushijima et al., 2021). Telomerase is silent in most normal somatic cells, but activated in 90% of cancer cells, making it an excellent target for cancer treatment. In the treatment of cancer, all kinds of telomerase activity inhibitors have failed due to their side effects. Coats plus (CP) is a rare autosomal recessive disease caused by CTC1 mutation, which is important for maintaining telomere length. CTC1L1142H mutation caused telomere damage. The point mutation of CTC1 using CRISPR/Cas9 technology confirmed that the interaction between CTC1 and STN1 is necessary to inhibit telomerase activity (Gu et al., 2018). Combining the biological functions of CRISPR/Cas9 and telomerase, the development of telomerase activating gene expression (Tage) has gradually become a new cancer gene therapy method. The Tage system consists of three components: the effector gene expression vector carrying 3‘telomerase recognition rod end, the dCas9-VP64 expression vector and the sgRNA artificial transcription factor expression vector targeting the telomere repeat sequence. Using AAV as a gene vector, the Tage system can effectively kill cancer cells and safely realize its application in the body (Dai et al., 2019). In cancer research using CRISPR system, CRISPR activation screening of targeted gRNA was carried out, gRNA libraries targeting different genes were established, targeted genes in cancer cells were systematically and accurately knocked out, and cancer gene therapy was achieved (Joung et al., 2022; Katti et al., 2022; Ye et al., 2022).

**FIGURE 5:**
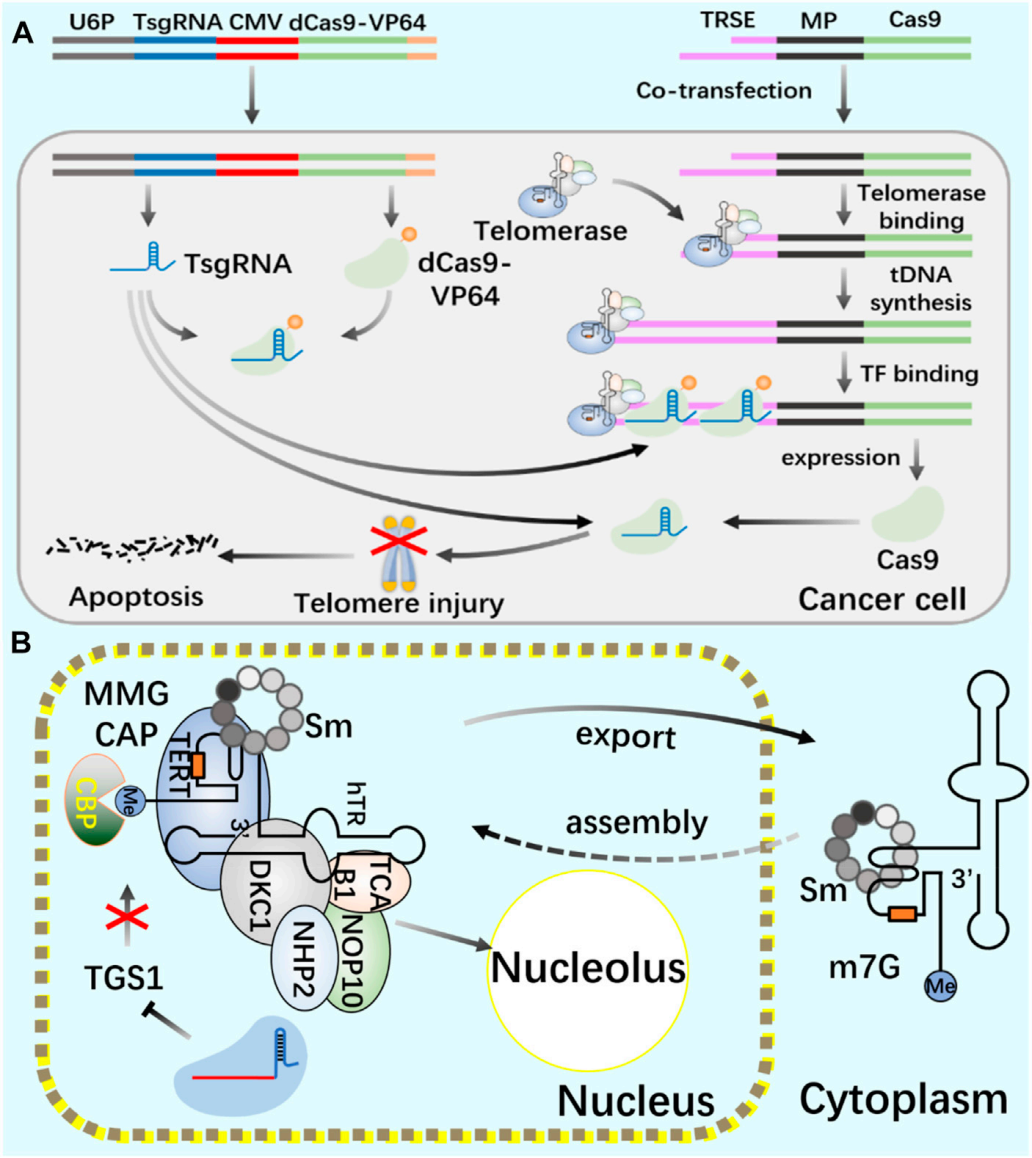
Application of CRISPR/Cas9 system in regulating telomerase and telomere **(A)** Use CRISPR/Cas9 and CRISPR/dCas9 systems to cut telomeres through telomerase to produce DNA damage and induce cancer cell death. **(B)** Use CRISPR/Cas9 system to introduce TGS1 gene frameshift mutation to realize the deletion of TGS1 hypermethylation enzyme and promote the increase of telomerase RNA and telomere elongation.

Telomerase activity usually depends on the expression level of TERT, which is the catalytic subunit of RNP complex (Barthel et al., 2017; Wu et al., 2021). The recruitment of telomerase to telomere occurs in the S phase of the cell cycle. By using CRISPR genome editing system and CRISPR-aided nano microscope technology to track telomerase in the nucleus, it is proved that telomerase uses three-dimensional diffusion to search for telomeres, and the recruitment of telomerase to telomere is driven by the dynamic interaction between the rapidly diffusing telomerase protein TERT and telomere protein TPP1 (Schmidt et al., 2016). In the study of human telomerase RNA (hTR) biogenic post-transcriptional modification, the use of CRISPR system consumes trimethyl guanosine synthetase 1 (TGS1). The reduction of trimethylation will increase the coupling of hTR with cap-binding complex (CBC) and Sec1/Munc-18 (Sm) chaperone protein, The accumulation of mature hTR in the nucleus and cytoplasm increases, and the increased hTR is assembled with TERT protein to produce increased active telomerase complex and increased telomerase activity, thus realizing the telomere elongation of cultured human cells. This study provides a new treatment scheme for telomerase dysfunction in telomeric syndrome (Figure 5) (Chen et al., 2020).

In order to further study the activation of telomerase and its activity regulation mechanism, in view of the low editing efficiency of CRISPR/Cas9 at the TERT gene locus, the genome editing method of “pop in/pop out” is used to realize precise modification of endogenous TERT gene sites in cells. This method provides a powerful tool for studying the biological function of telomerase using CRISPR/Cas9 (Kühn and Chu, 2015; Xi et al., 2015). Thus, the emergence of CRISPR system will provide an important tool for human research on telomerase and the regulation mechanism of cell aging.

## 6 dm^6^ACRISPR system and telomerase

**FIGURE 6:**
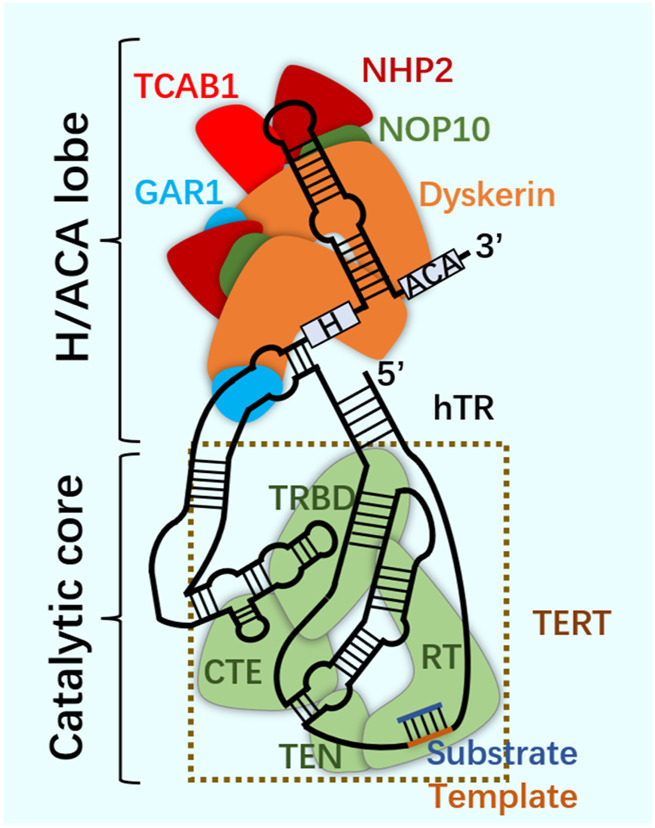
Telomerase.

**FIGURE 7:**
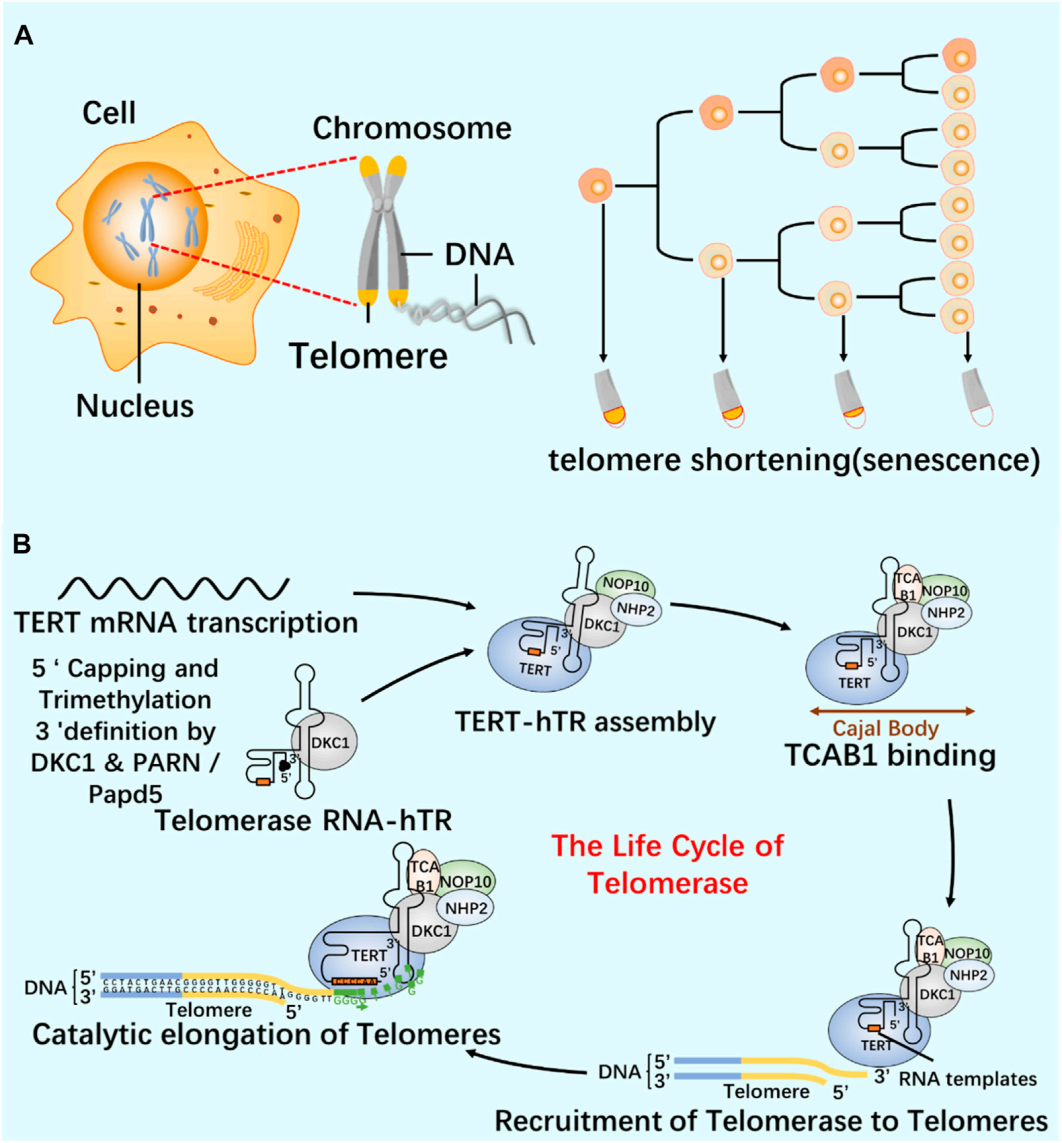
Relationship between telomeres and telomerase **(A)** Cell proliferation and telomere length reduction. Telomere is a repetitive DNA structure at the top of the chromosome. When the cell division DNA replicates, the telomere will protect the integrity of the chromosome. The activity of telomerase in normal cells was inhibited, and the telomere gradually shortened and disappeared with the continuous cell division. Chromosomes are finally completely exposed, cells cannot proliferate, DNA molecules degrade, and life ends. **(B)** The life cycle of telomerase and its regulation mechanism. Telomerase-protein RNA complex uses the non-coding RNA subunit hTR as a template, and the reverse transcriptase TERT catalyzes the telomere elongation. The life cycle of telomerase includes post-transcriptional modification (PTM) and maturation of hTR, intracellular localization, and effective assembly with TERT until the formation of a whole enzyme that can prolong telomeres.

As a repeat DNA sequence at the end of chromosome, telomere shortening is considered as a biological marker of cell aging (Al-Turki and Griffith, 2023). At each cell division, 50–100 pairs of base pairs will be lost in the chromosome end sequence, resulting in cell aging and even death (Blasco, 2005; Rossiello et al., 2022). Telomerase contains specialized TERT and telomerase RNA (TER), and has its own template and catalytic core required by TERT (Cash and Feigon, 2017; Jiang et al., 2018; Wang et al., 2019). In most human cancers, the increase of telomerase level makes cancer cells have the ability to proliferate indefinitely (Roake and Artandi, 2020). According to the characteristics of telomerase structure, composition and epigenetic modification (Figure 6; Figure 7), the telomere repeat sequence at the end of chromosome is extended to maintain the stability of genome, and the gradual loss of telomere caused by genome replication is offset. These are important for studying cell proliferation and delaying cell aging (He et al., 2021; Liu et al., 2022; Sekne et al., 2022).

The abnormal modification of RNA methylation is closely related to a series of cancer occurrence, and studying the relationship between m^6^A modification and tumor occurrence is of great significance for the treatment of cancer. In liver cancer research, it was found that methyltransferase METTL14 has a dual effect of promoting cancer cell proliferation and differentiation and inhibiting cancer cell metastasis (Ma et al., 2017; Chen et al., 2018); Overexpression of METTL5 promotes the growth, proliferation, migration, and invasion of liver cancer, knockdown of METTL5 promotes cell apoptosis, and inhibits the growth, proliferation, migration, and invasion of liver cancer (Peng et al., 2022). METTL3 has carcinogenic function in human liver cancer, and downregulation of METTL3 can weaken the tumorigenicity and lung metastasis of liver cancer (Chen and Wong, 2020). In glioblastoma, METTL3 can promote the maintenance and radiation resistance of glioblastoma stem cells and inhibit their self-renewal and proliferation (Cui et al., 2017; Visvanathan et al., 2018). Inhibition of FTO expression can hinder the growth, differentiation and self-renewal of glioblastoma stem cells (Cui et al., 2017). ALKBH5 can promote stem cell self-renewal and proliferation (Zhang et al., 2017). Overexpression of ALKBH5 was found in breast cancer research to enhance the enrichment of breast cancer stem cells (BCSC) (Zhang et al., 2016). In lung cancer and bladder cancer, METTL3 knockout can reduce the growth, survival and invasiveness of lung cancer cells, as well as the proliferation, invasion, *in vitro* survival and *in vivo* tumorigenicity of bladder cancer cells (Lin et al., 2016; Han et al., 2019). The m^6^A modification is closely related to the occurrence of cancer, and the m^6^A editing tool based on the CRISPR system will help to analyze the correlation mechanism between m^6^A modification and cancer occurrence.

At present, the dm^6^ACRISPR editing tool is constructed by combining the catalytically inactivated Cas protein with the m^6^A modification related protein (Li et al., 2020). This laid a foundation for studying the relationship between epigenetic modification and telomerase function and exploring the mechanism of m^6^A modification on telomerase activity regulation.

Telomerase structure. Telomerase is a ribonucleoprotein complex, which is composed of scaffold non-coding human telomerase RNA (hTR), telomerase reverse transcriptase (TERT) and related cofactors. Telomerase is composed of two RNA-linked structures. One is the H/ACA domain of hTR, which is composed of two groups of dyskerin complex (dyskerin, NHP2, NOP10 and GAR1) and TCAB1. The other contains the catalytic core, where hTR and TERT surround the telomere substrate. The two are connected through the CR4/5 domain of hTR.

### 6.1 Regulation of telomerase activity by m^6^A modification

**FIGURE 8:**
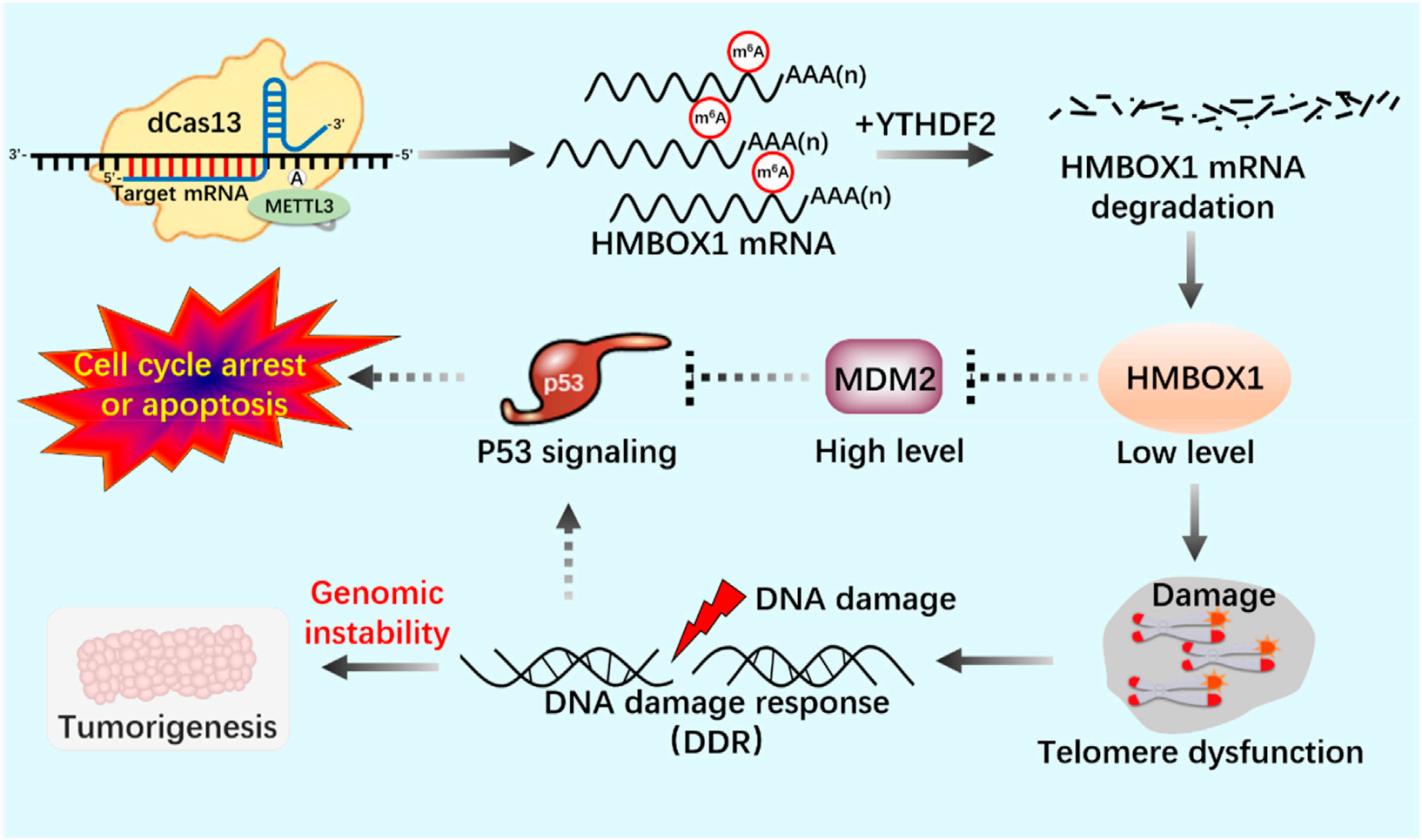
Application of m^6^A editing tool of dCas13b-METTL3 in telomerase activity regulation.

**FIGURE 9:**
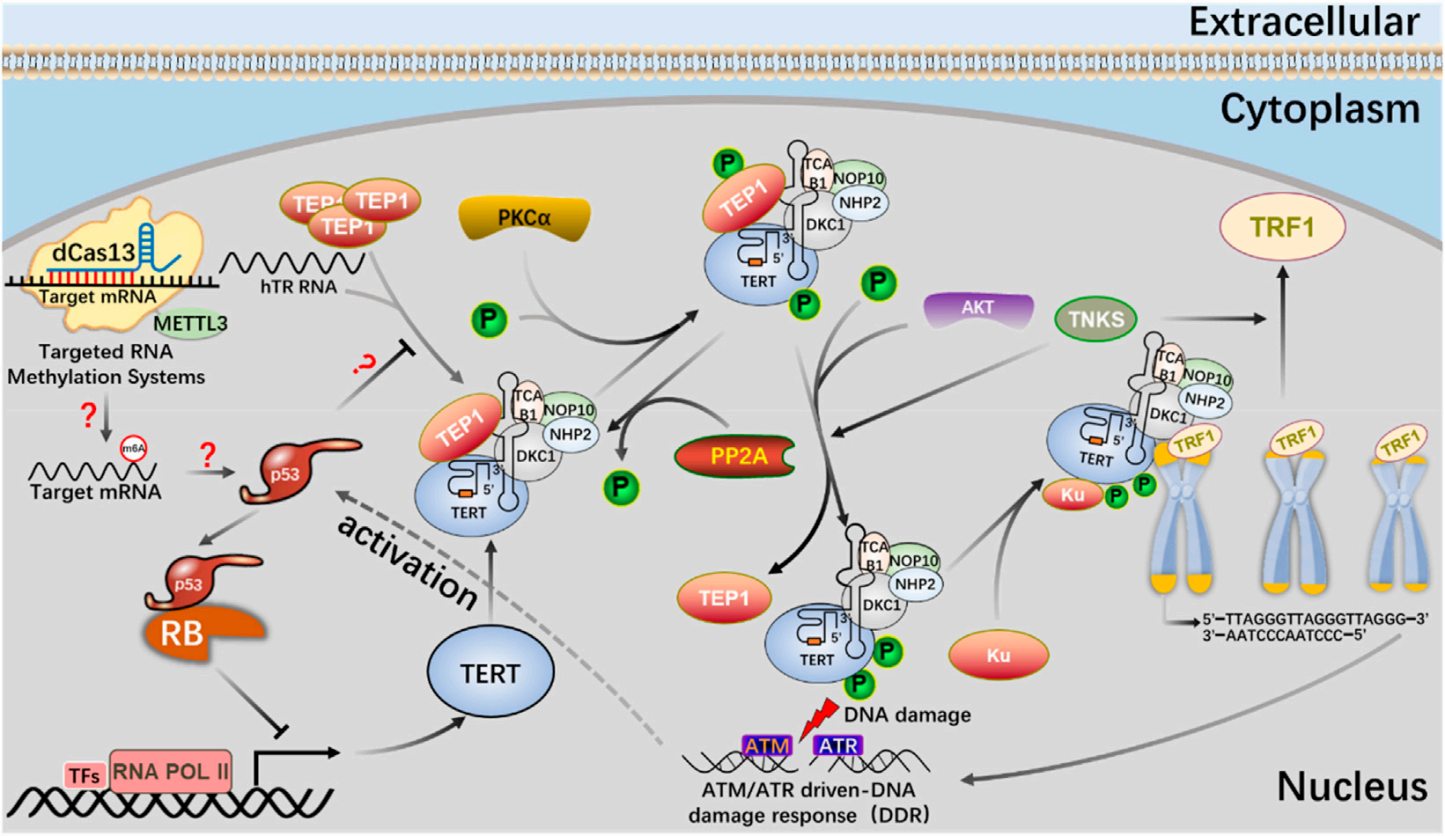
The mechanism of the m^6^A editing tool of CRISPR system to regulate telomerase activity and maintain telomere length in the p53 signal pathway.

RNA epigenetic modifications commonly include 5-methylcytidine (m^5^C) (Bohnsack et al., 2019), N6-methyladenosine (m^6^A) (Oerum et al., 2021), N7-methylguanosine (m^7^G) (Malbec et al., 2019), N1-methyladenosine (m^1^A) (Zhou et al., 2019), inosine (I) (Srinivasan et al., 2021), and pseudo uracil (Ψ) And dihydrouracil (D) (Haruehanroengra et al., 2020). m^6^A modification is closely related to many kinds of carcinogenesis, and altered m^6^A modification is widely involved in the progression of various tumorigenesis (Gu et al., 2020; Li et al., 2022). Deeply study m^6^A modification by regulating telomerase activity to maintain telomere homeostasis and genome stability is of great significance to clarify the role of m^6^A modification in cell aging and carcinogenesis (Table 2). Through Pan-Cancer Analysis of Whole Genomes (PCAWG) analysis of m^6^A modification of telomerase components, it was found that in most cancers, the expression level of telomerase components was positively correlated with methylase METTL3, negatively correlated with methylase METTL14, negatively correlated with demethylase FTO, negatively correlated with reading proteins YTHDC1, YTHDC2, YTHDF3 and FMR1, and positively correlated with reading proteins HNRNPC, HNRNP2B1, YTHDF1 and RBMX (Wang et al., 2023). These showed that there was a close relationship between telomerase component activity and m^6^A regulatory factors. With the help of the established CRISPR/dCas13 system to accurately edit the m^6^A modification platform, it is proved that the METTL3-HMBOX1 axis regulates telomere recruitment and telomere length related to telomerase in cancer cells, and leads to DNA damage reaction (Figure 8) (Lee et al., 2021). METTL3 promotes the stabilization of p53 protein and the expression of target genes in response to DNA damage and carcinogenic signals through catalytic activity dependent and independent mechanisms (Zhao et al., 2020; Raj et al., 2022). In addition, METTL3-m^6^A-p53 axis may be a potential target for the treatment of hepatocellular carcinoma (HCC) (Ke et al., 2022). Therefore, we can use CRISPR system to modify specific target genes with m^6^A, and regulate telomerase activity by regulating p53 signal pathway to maintain telomere homeostasis (Figure 9).

**TABLE 2:** Function of m^6^A regulator in telomerase activity.

m^6^A regulator	Key factor	Mechanism	Year [Ref]
METTL3	Cbf5	CircMEG3 relies on HULC to inhibit the expression of m^6^A methyltransferase METTL3, thus inhibiting the expression of Cbf5 and telomerase activity	2021 Jiang et al. (2021)
	HMBOX1	METTL3 overexpression mediates the downregulation of HMBOX1, which leads to telomere loss in cancer cells by interfering with the recruitment of telomerase complex	2021 Lee et al. (2021)
ALKBH5	Telomerase RNA (hTR)	Overexpression of ALKBH5 inhibits the assembly of TCAB1 and DKC1 in the telomerase structure by regulating the m^6^A modification in the H/ACA scaRNA domain of hTR, and inhibits telomerase activity	2020 Han et al. (2020)
YTHDF1	AGO2	The downregulation of YTHDF1 leads to abnormal deposition in AGO2 cytoplasm and the decrease of AGO2 content in nucleus, which destroys the relationship between TERT and TERC in the assembly of active telomerase RNP and inhibits telomerase activity	2019 Laudadio et al. (2019)
			2022 Li et al. (2022b)
HNRNP	hTERC	HNRNP F/H is overexpressed as a binding partner of hTERC and telomerase holoenzyme, activating telomerase and delaying stem cell aging	2021 Xu et al. (2020)

dCas13b-METTL3, a m^6^A editing tool based on CRISPR system, proves that METTL3-catalyzed HMBOX1 methylation is involved in regulating telomerase recruitment, resulting in telomere loss in cancer cells, and m^6^A is involved in carcinogenesis.

The m^6^A editing tool of CRISPR system modifies mRNA with m^6^A, affects telomerase activity through p53 signal pathway, participates in phosphorylation of PKC and AKT or dephosphorylation of PP2A, telomere shortening leads to DNA damage, and activates p53 signal pathway.

Through its reading protein, m^6^A modification is widely involved in biological processes such as pre-mRNA splicing, RNA output, mRNA translation and RNA degradation, and regulates the stability of targeted mRNA (He and He, 2021). In the study of m^6^A reading protein, it was found that proteins containing YTH domain (YTHDF1 and YTHDC1) used YTH domain to recognize m^6^A modification, YTHDF1 and YTHDF3 worked together to affect the translation of m^6^A containing mRNA, YTHDF2 accelerated the decay of mRNA, and YTHDC1 affected the nuclear processing of its target, further regulating the function and fate of m^6^A labeled mRNA (Roundtree et al., 2017b; Hsu et al., 2017; Shi et al., 2017).

**FIGURE 10:**
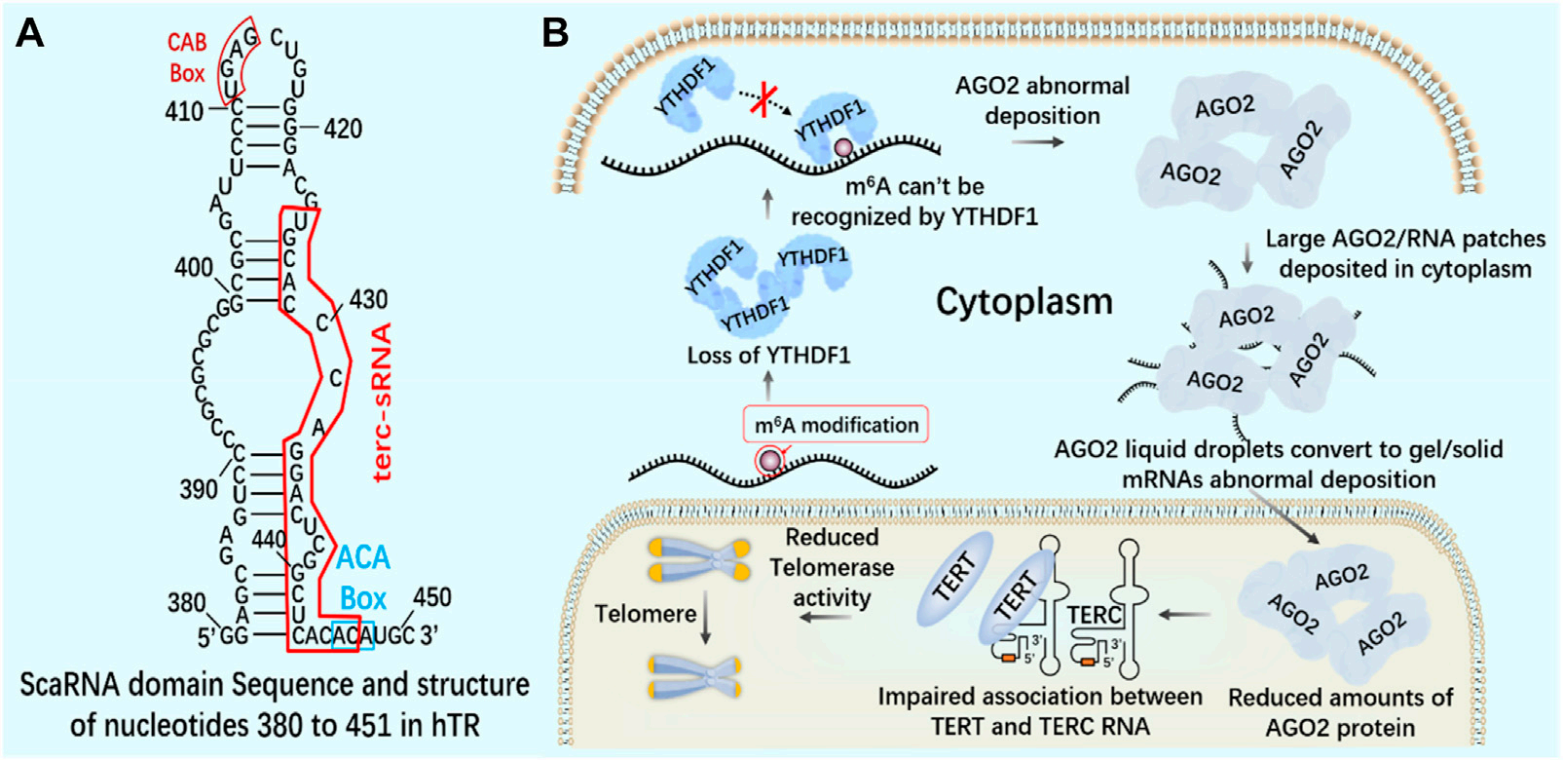
m^6^A reading protein and telomerase **(A)** The interaction between AGO2 and 23 nt sRNA produced by TTS of telomerase RNA component TERC (position 425–447) - terc-sRNA. **(B)** YTHDF1 interacts with AGO2 through YTH domain. YTHDF1 downregulates and destroys the interaction between YTHDF1 and AGO2. AGO2 deposits abnormally in the cytoplasm. AGO2 depletion destroys the association between TERT and TERC RNA, reduces telomerase activity, and leads to telomere shortening.

Knockout of YTHDF1 by CRISPR/Cas9 system will destroy the interaction between YT521-B homologous domain of YTHDF1 and AGO2 (argonaute 2), leading to the transformation of AGO2 droplets into gel/solids deposited in the cytoplasm (Li et al., 2022). In the nucleus, AGO2 interacts with 23 nt sRNA produced by TTS of telomerase RNA component telomerase RNA component (TERC) (position 425–447), which is called terc-sRNA. TERT and TERC constitute the core telomerase that maintains telomere length. As an RNA-binding protein, AGO2 has been found to promote telomerase activity and stimulate the association between TERT and TERC (Figure 10). AGO2 depletion leads to shorter telomeres and lower cell proliferation rate *in vitro* and *in vivo* (Laudadio et al., 2019). By regulating the recognition protein YTHDF1, it can regulate the consumption of AGO2 in the cytoplasm, affect the content of AGO2 in the nucleus, and lead to the change of telomerase activity in cells, which may lay the foundation for new therapeutic targets of tumor and telomeric diseases.

### 6.2 Site-directed modification of telomerase by dm^6^ACRISPR system

After CRISPR/Cas9 system, CRISPR/Cas13 system of type VI belongs to a known type that specifically binds and cleaves exogenous RNA (Abudayyeh et al., 2016; Shmakov et al., 2017; Smargon et al., 2017). CRISPR/Cas13 system can resist pathogenic RNA virus or regulate gene expression, and promote the knockout of mRNA, circular RNA and non-coding RNA (Wessels et al., 2020; Li et al., 2021). In addition, CRISPR/Cas13 system has been used for RNA modification *in vivo*, including editable regulation of selective splicing, A-to-I and C-to-U editing and m^6^A modification (O'Connell, 2019; Kordyś et al., 2022). Using CRISPR/Cas13 system, m^6^A can be added to specific RNA sites in a targeted way to achieve precise m^6^A modification at specific RNA sites. Since the methylation and demethylation process of m^6^A mainly occurs in the nucleus, two nuclear localization signal (NLS) peptides are added to dCasRx-METTL3 and dCasRx-ALKBH5 editors to realize the nuclear localization of the editing complex, which are called NLS-dCasRx-NLS-METTL3 and NLS-dCasRx-NLS-ALKBH5 (Xia et al., 2021). m^6^A methyltransferase METTL3 can increase the methylation modification level of telomerase related gene Cbf5 mRNA, promote its transcription and translation, and enhance telomerase activity (Jiang et al., 2021). As a nuclear protein reverse transcriptase, telomerase is composed of RNA template and catalytic protein (Wang et al., 2019). There is a 5-nt GGACU sequence with m^6^A common motif matching in the H/ACA scaRNA structure of hTR (Han et al., 2020), adenosine in the motif (A435) is located in the double stranded region of the RNA, suggesting that its secondary structure may be affected by m^6^A modification (Liu et al., 2015). The double stranded structure of the H/ACA scaRNA domain of hTR has been shown to be important for the assembly of telomerase complexes (Zhang et al., 2011). Overexpression of demethylase ALKBH5 leads to a decrease in the assembly efficiency of TCAB1 and DKC1 on telomerase, resulting in a decrease in telomerase activity. This may be mediated by modifying hTR to regulate telomerase assembly and function (Han et al., 2020).

**FIGURE 11:**
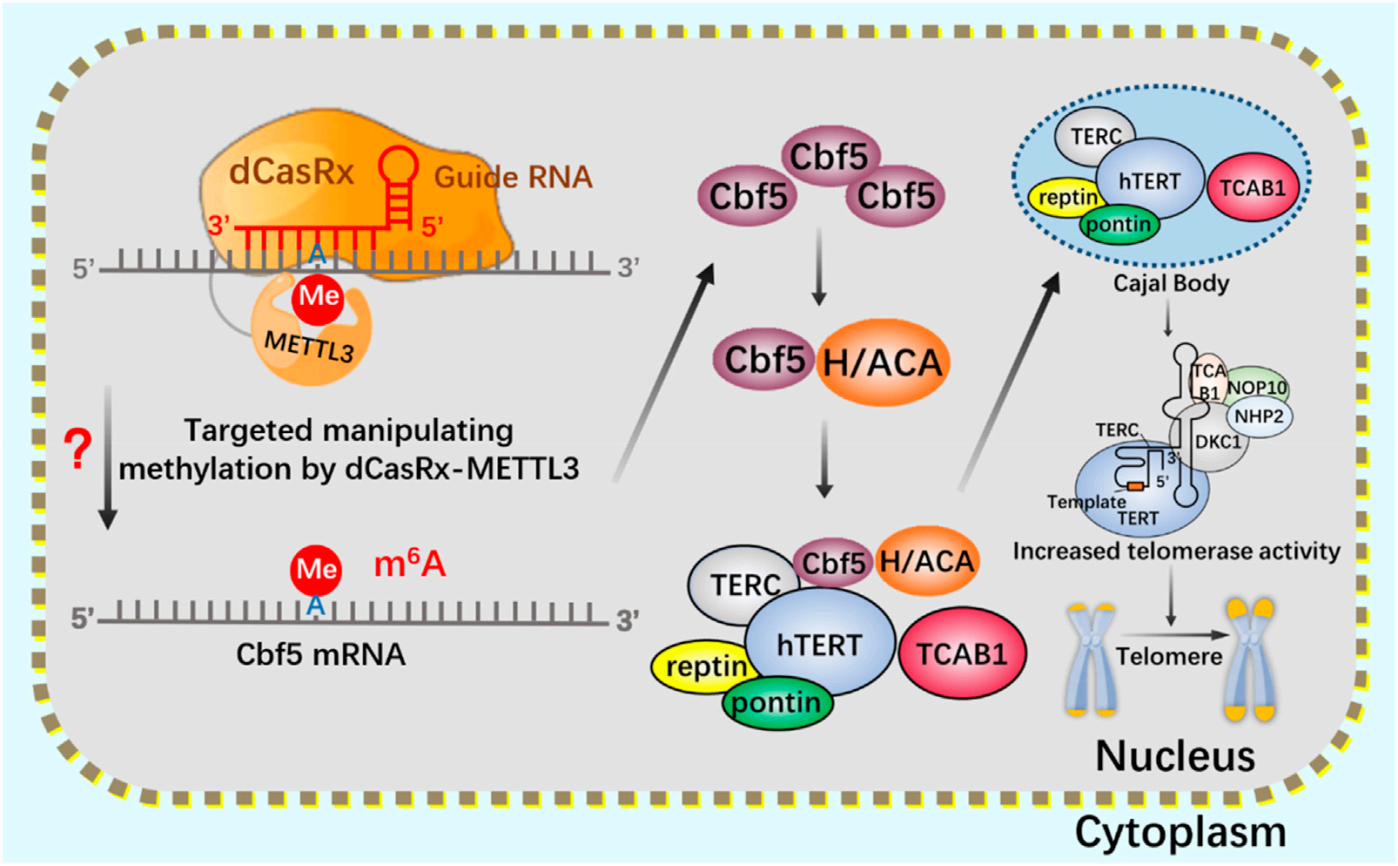
Regulation of telomere by m^6^A modification of NLS-dCasRx-NLS-METTL3 system.

**FIGURE 12:**
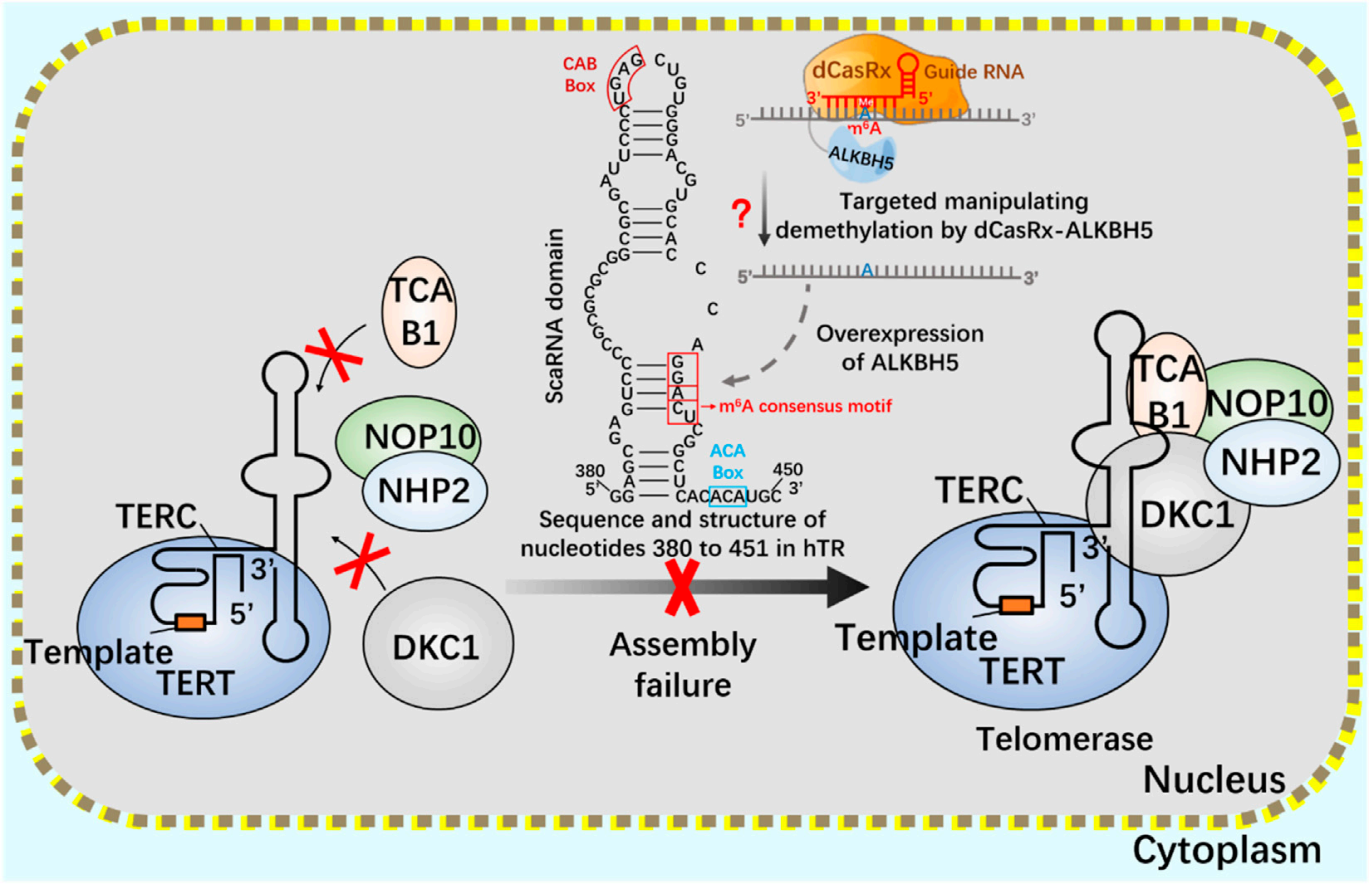
Regulation of telomerase assembly by m^6^A modification of NLS-dCasRx-NLS- ALKBH5 system m^6^A gene editing tool NLS-dCasRx-NLS-ALKBH5 locates dCasRx-ALKBH5 in the nucleus to achieve specific demethylation. Use the gene editing tool dCasRX-ALKBH5 to modify the m^6^A demethylation of telomerase hTR, regulate the assembly of telomerase components TCAB1 and DKC1, and reduce cell telomerase activity.

If telomerase activity is regulated by m6A modification, we consider attempting to achieve precise regulation using the nuclear localization CRISPR system combined with dCasRx and NLS. Assuming that the NLS-dCasRx-NLS-METTL3 system overexpressing METTL3 promotes Cbf5 transcription and translation (Figure 11), enhancing telomerase activity, and using NLS-dCasRx-NLS-ALKBH5 overexpressing ALKBH5 to remove m^6^A modification on hTR, Studying the regulation of TCAB1 and DKC1 assembly on telomerase by m^6^A modification (Figure 12) provides new insights into the potential application of CRISPR based m^6^A modification in telomerase regulation.

m^6^A gene editing tool NLS-dCasRx-NLS-METTL3 locates dCasRx-METTL3 in the nucleus to achieve specific methylation. Using the gene editing tool dCasRX-METTL3, the methylation modification level of Cbf5 mRNA was increased, the transcription and translation level of Cbf5 was enhanced, and Cbf5, as a component of telomere synthetase, increased telomere synthetase activity and regulated telomerase activity.

## 7 Conclusions and future prospects

CRISPR gene editing system, as the most revolutionary breakthrough in the field of biotechnology, is an unprecedented tool to cure human genetic diseases (Gillmore et al., 2021; Fox et al., 2022). m^6^A modification plays an important role in almost all-important biological processes (Liu et al., 2022; Boulias and Greer, 2022). Telomerase is highly active in stem cells, immune cells and germ cells to maintain telomere length (Jiang et al., 2018; Wan et al., 2021). Using CRISPR system to study the regulation mechanism of m^6^A modification on telomerase activity is of great significance for exploring the mechanism of cell proliferation and aging.

In this review, we systematically describe the latest application of CRISPR system in m^6^A modification and the regulation of telomerase activity, providing ideas for understanding the basic mechanism of regulating cell aging. When considering that m^6^A is the most common, frequent and conservative internal modification, and that telomerase activity is inhibited in normal cells, but remains high in most cancer cells, it is reasonable to propose that further exploring the mechanism of m^6^A modification on telomerase activity regulation will help to identify and develop gene therapy that can fight aging and treat cancer. It is now clear that the expression and activity of these proteins are essential for the correct regulation of the cell’s non-stop replication process. Strong evidence has emerged about the various functions of these proteins and the corresponding functions of targeted RNA in stem cells, immune cells, germ cells and sperm. So as we continue to decipher the epigenetic modification of m^6^A and the biology of cell proliferation and aging, we will have an important and in-depth understanding of the molecular mechanism of physiological and pathological cell aging.

## References

[B1] Abudayyeh O. O. Gootenberg J. S. Konermann S. Joung J. Slaymaker I. M. Cox D. B. (2016). C2c2 is a single-component programmable RNA-guided RNA-targeting CRISPR effector. Science 353 (6299), aaf5573. 10.1126/science.aaf5573 27256883 PMC5127784

[B2] Al-Turki T. M. Griffith J. D. (2023). Mammalian telomeric RNA (TERRA) can be translated to produce valine-arginine and glycine-leucine dipeptide repeat proteins. Proc. Natl. Acad. Sci. U. S. A. 120 (9), e2221529120. 10.1073/pnas.2221529120 36812212 PMC9992779

[B3] Anzalone A. V. Randolph P. B. Davis J. R. Sousa A. A. Koblan L. W. Levy J. M. (2019). Search-and-replace genome editing without double-strand breaks or donor DNA. Nature 576 (7785), 149–157. 10.1038/s41586-019-1711-4 31634902 PMC6907074

[B4] Bajaj S. Kumar M. S. Peters G. J. Mayur Y. C. (2020). Targeting telomerase for its advent in cancer therapeutics. Med. Res. Rev. 40 (5), 1871–1919. 10.1002/med.21674 32391613

[B5] Barthel F. P. Wei W. Tang M. Martinez-Ledesma E. Hu X. Amin S. B. (2017). Systematic analysis of telomere length and somatic alterations in 31 cancer types. Nat. Genet. 49 (3), 349–357. 10.1038/ng.3781 28135248 PMC5571729

[B6] Blackburn E. H. Collins K. (2011). Telomerase: An RNP enzyme synthesizes DNA. Cold Spring Harb. Perspect. Biol. 3 (5), 003558. 10.1101/cshperspect.a003558 PMC3101848 20660025

[B7] Blasco M. A. (2005). Telomeres and human disease: Ageing, cancer and beyond. Nat. Rev. Genet. 6 (8), 611–622. 10.1038/nrg1656 16136653

[B8] Bohnsack K. E. Höbartner C. Bohnsack M. T. (2019). Eukaryotic 5-methylcytosine (m⁵C) RNA methyltransferases: Mechanisms, cellular functions, and links to disease. Genes. (Basel) 10 (2), 102. 10.3390/genes10020102 30704115 PMC6409601

[B9] Boulias K. Greer E. L. (2022). Biological roles of adenine methylation in RNA. Nat. Rev. Genet. 24, 143–160. 10.1038/s41576-022-00534-0 36261710 PMC9974562

[B10] Cash D. D. Feigon J. (2017). Structure and folding of the Tetrahymena telomerase RNA pseudoknot. Nucleic Acids Res. 45 (1), 482–495. 10.1093/nar/gkw1153 27899638 PMC5224487

[B11] Celtikci B. Erkmen G. K. Dikmen Z. G. (2021). Regulation and effect of telomerase and telomeric length in stem cells. Curr. Stem Cell. Res. Ther. 16 (7), 809–823. 10.2174/1574888X15666200422104423 32321410

[B12] Chakravarti D. LaBella K. A. DePinho R. A. (2021). Telomeres: History, health, and hallmarks of aging. Cell. 184 (2), 306–322. 10.1016/j.cell.2020.12.028 33450206 PMC8081271

[B13] Chen L. Roake C. M. Galati A. Bavasso F. Micheli E. Saggio I. (2020). Loss of human TGS1 hypermethylase promotes increased telomerase RNA and telomere elongation. Cell. Rep. 30 (5), 1358–1372. 10.1016/j.celrep.2020.01.004 32023455 PMC7156301

[B14] Chen M. Wei L. Law C. T. Tsang F. H. Shen J. Cheng C. L. (2018). RNA N6-methyladenosine methyltransferase-like 3 promotes liver cancer progression through YTHDF2-dependent posttranscriptional silencing of SOCS2. Hepatology 67 (6), 2254–2270. 10.1002/hep.29683 29171881

[B15] Chen M. Wong C. M. (2020). The emerging roles of N6-methyladenosine (m6A) deregulation in liver carcinogenesis. Mol. Cancer 19 (1), 44. 10.1186/s12943-020-01172-y 32111216 PMC7047367

[B16] Chen X. Zhao Q. Zhao Y. L. Chai G. S. Cheng W. Zhao Z. (2021). Targeted RNA N(6) -methyladenosine demethylation controls cell fate transition in human pluripotent stem cells. Adv. Sci. (Weinh) 8 (11), 2003902. 10.1002/advs.202003902 34105279 PMC8188216

[B17] Chen Y. Yang S. Peng S. Li W. Wu F. Yao Q. (2019). N1-Methyladenosine detection with CRISPR-Cas13a/C2c2. Chem. Sci. 10 (10), 2975–2979. 10.1039/c8sc03408g 30996876 PMC6427938

[B18] Cong L. Ran F. A. Cox D. Lin S. Barretto R. Habib N. (2013). Multiplex genome engineering using CRISPR/Cas systems. Science 339 (6121), 819–823. 10.1126/science.1231143 23287718 PMC3795411

[B19] Cui Q. Shi H. Ye P. Li L. Qu Q. Sun G. (2017). m(6 A RNA methylation regulates the self-renewal and tumorigenesis of glioblastoma stem cells. Cell. Rep. 18 (11), 2622–2634. 10.1016/j.celrep.2017.02.059 28297667 PMC5479356

[B20] Dai W. Xu X. Wang D. Wu J. Wang J. (2019). Cancer therapy with a CRISPR-assisted telomerase-activating gene expression system. Oncogene 38 (21), 4110–4124. 10.1038/s41388-019-0707-8 30696954

[B21] Demanelis K. Jasmine F. Chen L. S. Chernoff M. Tong L. Delgado D. (2020). Determinants of telomere length across human tissues. Science 369 (6509), 6876. 10.1126/science.aaz6876 PMC8108546 32913074

[B22] Dogan F. Forsyth N. R. (2021). Epigenetic features in regulation of telomeres and telomerase in stem cells. Emerg. Top. Life Sci. 5 (4), 497–505. 10.1042/ETLS20200344 34486664

[B23] Dominissini D. Moshitch-Moshkovitz S. Schwartz S. Salmon-Divon M. Ungar L. Osenberg S. (2012). Topology of the human and mouse m6A RNA methylomes revealed by m6A-seq. Nature 485 (7397), 201–206. 10.1038/nature11112 22575960

[B24] Enache O. M. Rendo V. Abdusamad M. Lam D. Davison D. Pal S. (2020). Cas9 activates the p53 pathway and selects for p53-inactivating mutations. Nat. Genet. 52 (7), 662–668. 10.1038/s41588-020-0623-4 32424350 PMC7343612

[B25] Everette K. A. Newby G. A. Levine R. M. Mayberry K. Jang Y. Mayuranathan T. (2023). *Ex vivo* prime editing of patient haematopoietic stem cells rescues sickle-cell disease phenotypes after engraftment in mice. Nat. Biomed. Eng. 7, 616–628. 10.1038/s41551-023-01026-0 37069266 PMC10195679

[B26] Fischer N. W. Prodeus A. Malkin D. Gariépy J. (2016). p53 oligomerization status modulates cell fate decisions between growth, arrest and apoptosis. Cell. Cycle 15 (23), 3210–3219. 10.1080/15384101.2016.1241917 27754743 PMC5176156

[B27] Fox T. A. Houghton B. C. Petersone L. Waters E. Edner N. M. McKenna A. (2022). Therapeutic gene editing of T cells to correct CTLA-4 insufficiency. Sci. Transl. Med. 14 (668), eabn5811. 10.1126/scitranslmed.abn5811 36288278 PMC7617859

[B28] Ghaemi A. Bagheri E. Abnous K. Taghdisi S. M. Ramezani M. Alibolandi M. (2021). CRISPR-cas9 genome editing delivery systems for targeted cancer therapy. Life Sci. 267, 118969. 10.1016/j.lfs.2020.118969 33385410

[B29] Gillmore J. D. Gane E. Taubel J. Kao J. Fontana M. Maitland M. L. (2021). CRISPR-Cas9 *in vivo* gene editing for transthyretin amyloidosis. N. Engl. J. Med. 385 (6), 493–502. 10.1056/NEJMoa2107454 34215024

[B30] Gu C. Shi X. Dai C. Shen F. Rocco G. Chen J. (2020). RNA m(6)A modification in cancers: Molecular mechanisms and potential clinical applications. Innov. (Camb) 1 (3), 100066. 10.1016/j.xinn.2020.100066 PMC8454620 34557726

[B31] Gu P. Jia S. Takasugi T. Smith E. Nandakumar J. Hendrickson E. (2018). CTC1-STN1 coordinates G- and C-strand synthesis to regulate telomere length. Aging Cell. 17 (4), e12783. 10.1111/acel.12783 29774655 PMC6052479

[B32] Han J. Wang J. Z. Yang X. Yu H. Zhou R. Lu H. C. (2019). METTL3 promote tumor proliferation of bladder cancer by accelerating pri-miR221/222 maturation in m6A-dependent manner. Mol. Cancer 18 (1), 110. 10.1186/s12943-019-1036-9 31228940 PMC6588935

[B33] Han S. Zhao B. S. Myers S. A. Carr S. A. He C. Ting A. Y. (2020). RNA-protein interaction mapping via MS2- or Cas13-based APEX targeting. Proc. Natl. Acad. Sci. U. S. A. 117 (36), 22068–22079. 10.1073/pnas.2006617117 32839320 PMC7486720

[B34] Haruehanroengra P. Zheng Y. Y. Zhou Y. Huang Y. Sheng J. (2020). RNA modifications and cancer. RNA Biol. 17 (11), 1560–1575. 10.1080/15476286.2020.1722449 31994439 PMC7567502

[B35] He P. C. He C. (2021). m(6) A RNA methylation: from mechanisms to therapeutic potential. Embo J. 40 (3), e105977. 10.15252/embj.2020105977 33470439 PMC7849164

[B36] He Y. Wang Y. Liu B. Helmling C. Sušac L. Cheng R. (2021). Structures of telomerase at several steps of telomere repeat synthesis. Nature 593 (7859), 454–459. 10.1038/s41586-021-03529-9 33981033 PMC8857963

[B37] Höijer I. Emmanouilidou A. Östlund R. van Schendel R. Bozorgpana S. Tijsterman M. (2022). CRISPR-Cas9 induces large structural variants at on-target and off-target sites *in vivo* that segregate across generations. Nat. Commun. 13 (1), 627. 10.1038/s41467-022-28244-5 35110541 PMC8810904

[B38] Hsu P. D. Lander E. S. Zhang F. (2014). Development and applications of CRISPR-Cas9 for genome engineering. Cell. 157 (6), 1262–1278. 10.1016/j.cell.2014.05.010 24906146 PMC4343198

[B39] Hsu P. J. Zhu Y. Ma H. Guo Y. Shi X. Liu Y. (2017). Ythdc2 is an N(6)-methyladenosine binding protein that regulates mammalian spermatogenesis. Cell. Res. 27 (9), 1115–1127. 10.1038/cr.2017.99 28809393 PMC5587856

[B40] Hu Y. Gong C. Li Z. Liu J. Chen Y. Huang Y. (2022). Demethylase ALKBH5 suppresses invasion of gastric cancer via PKMYT1 m6A modification. Mol. Cancer 21 (1), 34. 10.1186/s12943-022-01522-y 35114989 PMC8812266

[B41] Ihry R. J. Worringer K. A. Salick M. R. Frias E. Ho D. Theriault K. (2018). p53 inhibits CRISPR-Cas9 engineering in human pluripotent stem cells. Nat. Med. 24 (7), 939–946. 10.1038/s41591-018-0050-6 29892062

[B42] Jiang J. Wang Y. Sušac L. Chan H. Basu R. Zhou Z. H. (2018). Structure of telomerase with telomeric DNA. Cell. 173 (5), 1179–1190. 10.1016/j.cell.2018.04.038 29775593 PMC5995583

[B43] Jiang X. Xing L. Chen Y. Qin R. Song S. Lu Y. (2021). CircMEG3 inhibits telomerase activity by reducing Cbf5 in human liver cancer stem cells. Mol. Ther. Nucleic Acids 23, 310–323. 10.1016/j.omtn.2020.11.009 33425489 PMC7779543

[B44] Jinek M. Chylinski K. Fonfara I. Hauer M. Doudna J. A. Charpentier E. (2012). A programmable dual-RNA-guided DNA endonuclease in adaptive bacterial immunity. Science 337 (6096), 816–821. 10.1126/science.1225829 22745249 PMC6286148

[B45] Joung J. Kirchgatterer P. C. Singh A. Cho J. H. Nety S. P. Larson R. C. (2022). CRISPR activation screen identifies BCL-2 proteins and B3GNT2 as drivers of cancer resistance to T cell-mediated cytotoxicity. Nat. Commun. 13 (1), 1606. 10.1038/s41467-022-29205-8 35338135 PMC8956604

[B46] Kantor A. McClements M. E. MacLaren R. E. (2020). CRISPR-Cas9 DNA base-editing and prime-editing. Int. J. Mol. Sci. 21 (17), 6240. 10.3390/ijms21176240 32872311 PMC7503568

[B47] Katti A. Diaz B. J. Caragine C. M. Sanjana N. E. Dow L. E. (2022). CRISPR in cancer biology and therapy. Nat. Rev. Cancer 22 (5), 259–279. 10.1038/s41568-022-00441-w 35194172

[B48] Ke W. Zhang L. Zhao X. Lu Z. (2022). p53 m(6 A modulation sensitizes hepatocellular carcinoma to apatinib through apoptosis. Apoptosis 27 (5-6), 426–440. 10.1007/s10495-022-01728-x 35503144

[B49] Killela P. J. Reitman Z. J. Jiao Y. Bettegowda C. Agrawal N. Diaz L. A. (2013). TERT promoter mutations occur frequently in gliomas and a subset of tumors derived from cells with low rates of self-renewal. Proc. Natl. Acad. Sci. U. S. A. 110 (15), 6021–6026. 10.1073/pnas.1303607110 23530248 PMC3625331

[B50] Kim D. Y. Moon S. B. Ko J. H. Kim Y. S. Kim D. (2020). Unbiased investigation of specificities of prime editing systems in human cells. Nucleic Acids Res. 48 (18), 10576–10589. 10.1093/nar/gkaa764 32941652 PMC7544197

[B51] Konermann S. Lotfy P. Brideau N. J. Oki J. Shokhirev M. N. Hsu P. D. (2018). Transcriptome engineering with RNA-targeting type VI-D CRISPR effectors. Cell. 173 (3), 665–676. 10.1016/j.cell.2018.02.033 29551272 PMC5910255

[B52] Kordyś M. Sen R. Warkocki Z. (2022). Applications of the versatile CRISPR-Cas13 RNA targeting system. Wiley Interdiscip. Rev. RNA 13 (3), 1694. 10.1002/wrna.1694 34553495

[B53] Kühn R. Chu V. T. (2015). Pop in, pop out: A novel gene-targeting strategy for use with CRISPR-cas9. Genome Biol. 16, 244. 10.1186/s13059-015-0810-2 26553112 PMC4640370

[B54] Kushawah G. Hernandez-Huertas L. Abugattas-Nuñez Del Prado J. Martinez-Morales J. R. DeVore M. L. Hassan H. (2020). CRISPR-Cas13d induces efficient mRNA knockdown in animal embryos. Dev. Cell. 54 (6), 805–817. 10.1016/j.devcel.2020.07.013 32768421

[B55] Laudadio I. Orso F. Azzalin G. Calabrò C. Berardinelli F. Coluzzi E. (2019). AGO2 promotes telomerase activity and interaction between the telomerase components TERT and TERC. EMBO Rep. 20 (2), 45969. 10.15252/embr.201845969 PMC6362350 30591524

[B56] Lee J. H. Hong J. Zhang Z. de la Peña Avalos B. Proietti C. J. Deamicis A. R. (2021). Regulation of telomere homeostasis and genomic stability in cancer by N (6)-adenosine methylation (m(6)A). Sci. Adv. 7 (31). 10.1126/sciadv.abg7073 PMC8318370 34321211

[B57] Leibowitz M. L. Papathanasiou S. Doerfler P. A. Blaine L. J. Sun L. Yao Y. (2021). Chromothripsis as an on-target consequence of CRISPR-Cas9 genome editing. Nat. Genet. 53 (6), 895–905. 10.1038/s41588-021-00838-7 33846636 PMC8192433

[B58] Li J. Chen K. Dong X. Xu Y. Sun Q. Wang H. (2022a). YTHDF1 promotes mRNA degradation via YTHDF1-AGO2 interaction and phase separation. Cell. Prolif. 55 (1), e13157. 10.1111/cpr.13157 34821414 PMC8780909

[B59] Li J. Chen Z. Chen F. Xie G. Ling Y. Peng Y. (2020a). Targeted mRNA demethylation using an engineered dCas13b-ALKBH5 fusion protein. Nucleic Acids Res. 48 (10), 5684–5694. 10.1093/nar/gkaa269 32356894 PMC7261189

[B60] Li S. Li X. Xue W. Zhang L. Yang L. Z. Cao S. M. (2021). Screening for functional circular RNAs using the CRISPR-Cas13 system. Nat. Methods 18 (1), 51–59. 10.1038/s41592-020-01011-4 33288960

[B61] Li X. Ma S. Deng Y. Yi P. Yu J. (2022b). Targeting the RNA m(6)A modification for cancer immunotherapy. Mol. Cancer 21 (1), 76. 10.1186/s12943-022-01558-0 35296338 PMC8924732

[B62] Li X. Qian X. Wang B. Xia Y. Zheng Y. Du L. (2020b). Programmable base editing of mutated TERT promoter inhibits brain tumour growth. Nat. Cell. Biol. 22 (3), 282–288. 10.1038/s41556-020-0471-6 32066906

[B63] Lin S. Choe J. Du P. Triboulet R. Gregory R. I. (2016). The m(6)A methyltransferase METTL3 promotes translation in human cancer cells. Mol. Cell. 62 (3), 335–345. 10.1016/j.molcel.2016.03.021 27117702 PMC4860043

[B64] Liu B. He Y. Wang Y. Song H. Zhou Z. H. Feigon J. (2022a). Structure of active human telomerase with telomere shelterin protein TPP1. Nature 604 (7906), 578–583. 10.1038/s41586-022-04582-8 35418675 PMC9912816

[B65] Liu L. Li H. Hu D. Wang Y. Shao W. Zhong J. (2022b). Insights into N6-methyladenosine and programmed cell death in cancer. Mol. Cancer 21 (1), 32. 10.1186/s12943-022-01508-w 35090469 PMC8796496

[B66] Liu N. Dai Q. Zheng G. He C. Parisien M. Pan T. (2015). N(6)-methyladenosine-dependent RNA structural switches regulate RNA-protein interactions. Nature 518 (7540), 560–564. 10.1038/nature14234 25719671 PMC4355918

[B67] Liu X. M. Zhou J. Mao Y. Ji Q. Qian S. B. (2019). Programmable RNA N(6)-methyladenosine editing by CRISPR-Cas9 conjugates. Nat. Chem. Biol. 15 (9), 865–871. 10.1038/s41589-019-0327-1 31383972 PMC6702037

[B68] Liu Z. Liao Z. Chen Y. Han L. Yin Q. Xiao H. (2020). Application of various delivery methods for CRISPR/dCas9. Mol. Biotechnol. 62 (8), 355–363. 10.1007/s12033-020-00258-8 32583364

[B69] Lupatov A. Y. Yarygin K. N. (2022). Telomeres and telomerase in the control of stem cells. Biomedicines 10 (10), 2335. 10.3390/biomedicines10102335 36289597 PMC9598777

[B70] Ma J. Z. Yang F. Zhou C. C. Liu F. Yuan J. H. Wang F. (2017). METTL14 suppresses the metastatic potential of hepatocellular carcinoma by modulating N(6) -methyladenosine-dependent primary MicroRNA processing. Hepatology 65 (2), 529–543. 10.1002/hep.28885 27774652

[B71] Malbec L. Zhang T. Chen Y. S. Zhang Y. Sun B. F. Shi B. Y. (2019). Dynamic methylome of internal mRNA N(7)-methylguanosine and its regulatory role in translation. Cell. Res. 29 (11), 927–941. 10.1038/s41422-019-0230-z 31520064 PMC6889513

[B72] Manghwar H. Lindsey K. Zhang X. Jin S. (2019). CRISPR/Cas system: Recent advances and future prospects for genome editing. Trends Plant Sci. 24 (12), 1102–1125. 10.1016/j.tplants.2019.09.006 31727474

[B73] Negrini S. De Palma R. Filaci G. (2020). Anti-cancer immunotherapies targeting telomerase. Cancers (Basel) 12 (8), 2260. 10.3390/cancers12082260 32806719 PMC7465444

[B74] O'Connell M. R. (2019). Molecular mechanisms of RNA targeting by cas13-containing type VI CRISPR-cas systems. J. Mol. Biol. 431 (1), 66–87. 10.1016/j.jmb.2018.06.029 29940185

[B75] Oerum S. Meynier V. Catala M. Tisné C. (2021). A comprehensive review of m6A/m6Am RNA methyltransferase structures. Nucleic Acids Res. 49 (13), 7239–7255. 10.1093/nar/gkab378 34023900 PMC8287941

[B76] Peng H. Chen B. Wei W. Guo S. Han H. Yang C. (2022). N(6)-methyladenosine (m(6)A) in 18S rRNA promotes fatty acid metabolism and oncogenic transformation. Nat. Metab. 4 (8), 1041–1054. 10.1038/s42255-022-00622-9 35999469

[B77] Raj N. Wang M. Seoane J. A. Zhao R. L. Kaiser A. M. Moonie N. A. (2022). The Mettl3 epitranscriptomic writer amplifies p53 stress responses. Mol. Cell. 82 (13), 2370–2384. 10.1016/j.molcel.2022.04.010 35512709 PMC9807187

[B78] Roake C. M. Artandi S. E. (2020). Regulation of human telomerase in homeostasis and disease. Nat. Rev. Mol. Cell. Biol. 21 (7), 384–397. 10.1038/s41580-020-0234-z 32242127 PMC7377944

[B79] Rossiello F. Jurk D. Passos J. F. d'Adda di Fagagna F. (2022). Telomere dysfunction in ageing and age-related diseases. Nat. Cell. Biol. 24 (2), 135–147. 10.1038/s41556-022-00842-x 35165420 PMC8985209

[B80] Roundtree I. A. Evans M. E. Pan T. He C. (2017a). Dynamic RNA modifications in gene expression regulation. Cell. 169 (7), 1187–1200. 10.1016/j.cell.2017.05.045 28622506 PMC5657247

[B81] Roundtree I. A. Luo G. Z. Zhang Z. Wang X. Zhou T. Cui Y. (2017b). YTHDC1 mediates nuclear export of N(6)-methyladenosine methylated mRNAs. Elife 6, 31311. 10.7554/eLife.31311 PMC5648532 28984244

[B82] Schmidt J. C. Zaug A. J. Cech T. R. (2016). Live cell imaging reveals the dynamics of telomerase recruitment to telomeres. Cell. 166 (5), 1188–1197. 10.1016/j.cell.2016.07.033 27523609 PMC5743434

[B83] Schmidt W. Arnold H. H. Kersten H. (1975). Biosynthetic pathway of ribothymidine in B. subtilis and M. lysodeikticus involving different coenzymes for transfer RNA and ribosomal RNA. Nucleic Acids Res. 2 (7), 1043–1051. 10.1093/nar/2.7.1043 807911 PMC343492

[B84] Sekne Z. Ghanim G. E. van Roon A. M. Nguyen T. H. D. (2022). Structural basis of human telomerase recruitment by TPP1-POT1. Science 375 (6585), 1173–1176. 10.1126/science.abn6840 35201900 PMC7612489

[B85] Shay J. W. (2016). Role of telomeres and telomerase in aging and cancer. Cancer Discov. 6 (6), 584–593. 10.1158/2159-8290.CD-16-0062 27029895 PMC4893918

[B86] Shi H. Wang X. Lu Z. Zhao B. S. Ma H. Hsu P. J. (2017). YTHDF3 facilitates translation and decay of N(6)-methyladenosine-modified RNA. Cell. Res. 27 (3), 315–328. 10.1038/cr.2017.15 28106072 PMC5339834

[B87] Shi H. Z. Xiong J. S. Gan L. Zhang Y. Zhang C. C. Kong Y. Q. (2022). N6-methyladenosine reader YTHDF3 regulates melanoma metastasis via its 'executor'LOXL3. Clin. Transl. Med. 12 (11), 1075. 10.1002/ctm2.1075 PMC9630608 36324258

[B88] Shmakov S. Smargon A. Scott D. Cox D. Pyzocha N. Yan W. (2017). Diversity and evolution of class 2 CRISPR-Cas systems. Nat. Rev. Microbiol. 15 (3), 169–182. 10.1038/nrmicro.2016.184 28111461 PMC5851899

[B89] Sinha S. Barbosa K. Cheng K. Leiserson M. D. M. Jain P. Deshpande A. (2021). A systematic genome-wide mapping of oncogenic mutation selection during CRISPR-Cas9 genome editing. Nat. Commun. 12 (1), 6512. 10.1038/s41467-021-26788-6 34764240 PMC8586238

[B90] Smargon A. A. Cox D. B. T. Pyzocha N. K. Zheng K. Slaymaker I. M. Gootenberg J. S. (2017). Cas13b is a type VI-B CRISPR-associated RNA-guided RNase differentially regulated by accessory proteins Csx27 and Csx28. Mol. Cell. 65 (4), 618–630. 10.1016/j.molcel.2016.12.023 28065598 PMC5432119

[B91] Srinivasan S. Torres A. G. Ribas de Pouplana L. (2021). Inosine in biology and disease. Genes. (Basel) 12 (4), 600. 10.3390/genes12040600 33921764 PMC8072771

[B92] Sun L. Chiang J. Y. Choi J. Y. Xiong Z. M. Mao X. Collins F. S. (2019). Transient induction of telomerase expression mediates senescence and reduces tumorigenesis in primary fibroblasts. Proc. Natl. Acad. Sci. U. S. A. 116 (38), 18983–18993. 10.1073/pnas.1907199116 31481614 PMC6754593

[B93] Tang C. Xie Y. Yu T. Liu N. Wang Z. Woolsey R. J. (2020). m(6 A-dependent biogenesis of circular RNAs in male germ cells. Cell. Res. 30 (3), 211–228. 10.1038/s41422-020-0279-8 32047269 PMC7054367

[B94] Trybek T. Kowalik A. Góźdź S. Kowalska A. (2020). Telomeres and telomerase in oncogenesis. Oncol. Lett. 20 (2), 1015–1027. 10.3892/ol.2020.11659 32724340 PMC7377093

[B95] Ushijima T. Clark S. J. Tan P. (2021). Mapping genomic and epigenomic evolution in cancer ecosystems. Science 373 (6562), 1474–1479. 10.1126/science.abh1645 34554797

[B96] Visvanathan A. Patil V. Arora A. Hegde A. S. Arivazhagan A. Santosh V. (2018). Essential role of METTL3-mediated m(6)A modification in glioma stem-like cells maintenance and radioresistance. Oncogene 37 (4), 522–533. 10.1038/onc.2017.351 28991227

[B97] Wan F. Ding Y. Zhang Y. Wu Z. Li S. Yang L. (2021). Zipper head mechanism of telomere synthesis by human telomerase. Cell. Res. 31 (12), 1275–1290. 10.1038/s41422-021-00586-7 34782750 PMC8648750

[B98] Wang J. Dai M. Xing X. Wang X. Qin X. Huang T. (2023). Genomic, epigenomic, and transcriptomic signatures for telomerase complex components: A pan-cancer analysis. Mol. Oncol. 17 (1), 150–172. 10.1002/1878-0261.13324 36239411 PMC9812836

[B99] Wang S. Guo M. Zhu Y. Lin Z. Huang Z. (2022). Cryo-EM structure of the type III-E CRISPR-Cas effector gRAMP in complex with TPR-CHAT. Cell. Res. 32, 1128–1131. 10.1038/s41422-022-00738-3 36280712 PMC9715532

[B100] Wang Y. Sušac L. Feigon J. (2019). Structural biology of telomerase. Cold Spring Harb. Perspect. Biol. 11 (12), 032383. 10.1101/cshperspect.a032383 PMC6886448 31451513

[B101] Wei J. Yu X. Yang L. Liu X. Gao B. Huang B. (2022). FTO mediates LINE1 m(6)A demethylation and chromatin regulation in mESCs and mouse development. Science 376 (6596), 968–973. 10.1126/science.abe9582 35511947 PMC9746489

[B102] Wessels H. H. Méndez-Mancilla A. Guo X. Legut M. Daniloski Z. Sanjana N. E. (2020). Massively parallel Cas13 screens reveal principles for guide RNA design. Nat. Biotechnol. 38 (6), 722–727. 10.1038/s41587-020-0456-9 32518401 PMC7294996

[B103] Wilson C. Chen P. J. Miao Z. Liu D. R. (2020). Programmable m(6)A modification of cellular RNAs with a Cas13-directed methyltransferase. Nat. Biotechnol. 38 (12), 1431–1440. 10.1038/s41587-020-0572-6 32601430 PMC7718427

[B104] Wu L. Fidan K. Um J. Y. Ahn K. S. (2020). Telomerase: Key regulator of inflammation and cancer. Pharmacol. Res. 155, 104726. 10.1016/j.phrs.2020.104726 32109579

[B105] Wu X. Wu J. Dai J. Chen B. Chen Z. Wang S. (2021). Aggregation-induced emission luminogens reveal cell cycle-dependent telomerase activity in cancer cells. Natl. Sci. Rev. 8 (6), 306. 10.1093/nsr/nwaa306 PMC8288165 34691667

[B106] Wu Y. Chen Z. Xie G. Zhang H. Wang Z. Zhou J. (2022). RNA m(1)A methylation regulates glycolysis of cancer cells through modulating ATP5D. Proc. Natl. Acad. Sci. U. S. A. 119 (28), 2119038119. 10.1073/pnas.2119038119 PMC9282374 35867754

[B107] Xi L. Schmidt J. C. Zaug A. J. Ascarrunz D. R. Cech T. R. (2015). A novel two-step genome editing strategy with CRISPR-Cas9 provides new insights into telomerase action and TERT gene expression. Genome Biol. 16, 231. 10.1186/s13059-015-0791-1 26553065 PMC4640169

[B108] Xia Z. Tang M. Ma J. Zhang H. Gimple R. C. Prager B. C. (2021). Epitranscriptomic editing of the RNA N6-methyladenosine modification by dCasRx conjugated methyltransferase and demethylase. Nucleic Acids Res. 49 (13), 7361–7374. 10.1093/nar/gkab517 34181729 PMC8287920

[B109] Xie S. Jin H. Yang F. Zheng H. Chang Y. Liao Y. (2021). Programmable RNA N(1) -methyladenosine demethylation by a cas13d-directed demethylase. Angew. Chem. Int. Ed. Engl. 60 (36), 19592–19597. 10.1002/anie.202105253 34081827

[B110] Xu C. Xie N. Su Y. Sun Z. Liang Y. Zhang N. (2020). HnRNP F/H associate with hTERC and telomerase holoenzyme to modulate telomerase function and promote cell proliferation. Cell. Death Differ. 27 (6), 1998–2013. 10.1038/s41418-019-0483-6 31863069 PMC7244589

[B111] Yang L. Z. Wang Y. Li S. Q. Yao R. W. Luan P. F. Wu H. (2019). Dynamic imaging of RNA in living cells by CRISPR-cas13 systems. Mol. Cell. 76 (6), 981–997. 10.1016/j.molcel.2019.10.024 31757757

[B112] Yang Y. Hsu P. J. Chen Y. S. Yang Y. G. (2018). Dynamic transcriptomic m(6)A decoration: Writers, erasers, readers and functions in RNA metabolism. Cell. Res. 28 (6), 616–624. 10.1038/s41422-018-0040-8 29789545 PMC5993786

[B113] Ye L. Park J. J. Peng L. Yang Q. Chow R. D. Dong M. B. (2022). A genome-scale gain-of-function CRISPR screen in CD8 T cells identifies proline metabolism as a means to enhance CAR-T therapy. Cell. Metab. 34 (4), 595–614. 10.1016/j.cmet.2022.02.009 35276062 PMC8986623

[B114] Ying X. Jiang X. Zhang H. Liu B. Huang Y. Zhu X. (2020). Programmable N6-methyladenosine modification of CDCP1 mRNA by RCas9-methyltransferase like 3 conjugates promotes bladder cancer development. Mol. Cancer 19 (1), 169. 10.1186/s12943-020-01289-0 33267838 PMC7712571

[B115] Yu G. Kim H. K. Park J. Kwak H. Cheong Y. Kim D. (2023). Prediction of efficiencies for diverse prime editing systems in multiple cell types. Cell. 186 (10), 2256–2272. 10.1016/j.cell.2023.03.034 37119812

[B116] Yu G. Wang X. Zhang Y. An Q. Wen Y. Li X. (2022). Structure and function of a bacterial type III-E CRISPR-Cas7-11 complex. Nat. Microbiol. 7, 2078–2088. 10.1038/s41564-022-01256-z 36302881

[B117] Zhan T. Rindtorff N. Betge J. Ebert M. P. Boutros M. (2019). CRISPR/Cas9 for cancer research and therapy. Semin. Cancer Biol. 55, 106–119. 10.1016/j.semcancer.2018.04.001 29673923

[B118] Zhang C. Konermann S. Brideau N. J. Lotfy P. Wu X. Novick S. J. (2018). Structural basis for the RNA-guided ribonuclease activity of CRISPR-cas13d. Cell. 175 (1), 212–223.e17. 10.1016/j.cell.2018.09.001 30241607 PMC6179368

[B119] Zhang C. Samanta D. Lu H. Bullen J. W. Zhang H. Chen I. (2016). Hypoxia induces the breast cancer stem cell phenotype by HIF-dependent and ALKBH5-mediated m⁶A-demethylation of NANOG mRNA. Proc. Natl. Acad. Sci. U. S. A. 113 (14), E2047–E2056. 10.1073/pnas.1602883113 27001847 PMC4833258

[B120] Zhang H. Qin C. An C. Zheng X. Wen S. Chen W. (2021). Application of the CRISPR/Cas9-based gene editing technique in basic research, diagnosis, and therapy of cancer. Mol. Cancer 20 (1), 126. 10.1186/s12943-021-01431-6 34598686 PMC8484294

[B121] Zhang Q. Kim N. K. Feigon J. (2011). Architecture of human telomerase RNA. Proc. Natl. Acad. Sci. U. S. A. 108 (51), 20325–20332. 10.1073/pnas.1100279108 21844345 PMC3251123

[B122] Zhang S. Zhao B. S. Zhou A. Lin K. Zheng S. Lu Z. (2017). m(6 A demethylase ALKBH5 maintains tumorigenicity of glioblastoma stem-like cells by sustaining FOXM1 expression and cell proliferation program. Cancer Cell. 31 (4), 591–606.e6. 10.1016/j.ccell.2017.02.013 28344040 PMC5427719

[B123] Zhao T. Sun D. Zhao M. Lai Y. Liu Y. Zhang Z. (2020). N(6)-methyladenosine mediates arsenite-induced human keratinocyte transformation by suppressing p53 activation. Environ. Pollut. 259, 113908. 10.1016/j.envpol.2019.113908 31931413 PMC7082205

[B124] Zhou C. Hu X. Tang C. Liu W. Wang S. Zhou Y. (2020). CasRx-mediated RNA targeting prevents choroidal neovascularization in a mouse model of age-related macular degeneration. Natl. Sci. Rev. 7 (5), 835–837. 10.1093/nsr/nwaa033 34692105 PMC8288881

[B125] Zhou H. Rauch S. Dai Q. Cui X. Zhang Z. Nachtergaele S. (2019). Evolution of a reverse transcriptase to map N(1)-methyladenosine in human messenger RNA. Nat. Methods 16 (12), 1281–1288. 10.1038/s41592-019-0550-4 31548705 PMC6884687

